# The kinetoplastid kinetochore protein KKT4 is an unconventional microtubule tip–coupling protein

**DOI:** 10.1083/jcb.201711181

**Published:** 2018-11-05

**Authors:** Aida Llauró, Hanako Hayashi, Megan E. Bailey, Alex Wilson, Patryk Ludzia, Charles L. Asbury, Bungo Akiyoshi

**Affiliations:** 1 Department of Physiology and Biophysics, University of Washington, Seattle, WA; 2 Department of Biochemistry, University of Oxford, Oxford, UK

## Abstract

Kinetochores are multiprotein machines that drive chromosome segregation by maintaining persistent, load-bearing linkages between chromosomes and dynamic microtubule tips. Kinetochores in commonly studied eukaryotes bind microtubules through widely conserved components like the Ndc80 complex. However, in evolutionarily divergent kinetoplastid species such as *Trypanosoma brucei*, which causes sleeping sickness, the kinetochores assemble from a unique set of proteins lacking homology to any known microtubule-binding domains. Here, we show that the *T. brucei* kinetochore protein KKT4 binds directly to microtubules and maintains load-bearing attachments to both growing and shortening microtubule tips. The protein localizes both to kinetochores and to spindle microtubules in vivo, and its depletion causes defects in chromosome segregation. We define a microtubule-binding domain within KKT4 and identify several charged residues important for its microtubule-binding activity. Thus, despite its lack of significant similarity to other known microtubule-binding proteins, KKT4 has key functions required for driving chromosome segregation. We propose that it represents a primary element of the kinetochore–microtubule interface in kinetoplastids.

## Abstract (precis)

The evolutionarily divergent class of kinetoplastid organisms has a set of unconventional kinetochore proteins that drive chromosome segregation, but it is unclear which components interact with spindle microtubules. Llauró et al. now identify KKT4 as the first microtubule-binding kinetochore protein in *Trypanosoma brucei*, a major human pathogenic parasite.

## Introduction

Chromosome segregation in eukaryotes depends on the interaction between chromosomes and dynamic spindle microtubules (McIntosh, 2016). The interaction is mediated by the macromolecular kinetochore complex that assembles onto the centromeric region of each chromosome (Cheeseman, 2014; Musacchio and Desai, 2017). Spindle microtubules are dynamic polymers that grow and shrink by addition and loss of tubulin subunits from their tips (Desai and Mitchison, 1997). Accurate chromosome segregation requires that kinetochores maintain persistent, load-bearing attachments to dynamic microtubule tips, even as the tips assemble and disassemble under their grip (Joglekar et al., 2010; McIntosh, 2017).

The kinetochore consists of more than 30 structural proteins, even in budding yeast, which has relatively simple kinetochores (Biggins, 2013). Among these components, CENP-A is a centromere-specific histone H3 variant that forms a specialized chromatin environment at the centromere, while the Ndc80 complex directly binds microtubules to mediate the coupling of kinetochores to microtubule tips (Musacchio and Desai, 2017). The Ndc80 complex consists of the Ndc80, Nuf2, Spc24, and Spc25 proteins (Wigge and Kilmartin, 2001). Microtubule-binding activities reside in the Ndc80 and Nuf2 proteins, which both carry calponin homology (CH) domains (Cheeseman et al., 2006; Wei et al., 2007; Ciferri et al., 2008). Ensembles of Ndc80 complexes can form load-bearing attachments to dynamic microtubule tips in vitro (McIntosh et al., 2008; Powers et al., 2009; Umbreit et al., 2012; Volkov et al., 2018). Besides Ndc80, there are several additional microtubule-binding components that localize at kinetochores, including the Dam1 complex and Stu2 protein in yeasts, and the Ska complex in metazoa (Cheeseman et al., 2001; He et al., 2001; Tanaka et al., 2005; Hanisch et al., 2006; Hsu and Toda, 2011). These complexes are also capable of forming dynamic, load-bearing attachments in vitro (Asbury et al., 2006; Westermann et al., 2006; Grishchuk et al., 2008; Welburn et al., 2009; Miller et al., 2016). Extensive studies have been performed to understand the nature of their microtubule-binding properties (Miranda et al., 2005; Wei et al., 2005; Al-Bassam et al., 2006; Cheeseman et al., 2006; Westermann et al., 2006; Ciferri et al., 2008; Welburn et al., 2009; Tien et al., 2010; Ayaz et al., 2012; Jeyaprakash et al., 2012; Schmidt et al., 2012; Umbreit et al., 2014; Janczyk et al., 2017; Kim et al., 2017; Maciejowski et al., 2017).

Putative homologues of CENP-A and Ndc80 complex components are found in various eukaryotes sequenced thus far (Meraldi et al., 2006; Drinnenberg and Akiyoshi, 2017; van Hooff et al., 2017). However, neither these nor any of the other conventional kinetochore proteins have been found in kinetoplastids, a group of evolutionarily divergent eukaryotes including the parasitic trypanosomatids, which are responsible for sleeping sickness (*Trypanosoma brucei*), Chagas disease (*Trypanosoma cruzi*), and leishmaniasis (*Leishmania* species; Berriman et al., 2005; El-Sayed et al., 2005; Ivens et al., 2005). Using a YFP-tagging screen and mass spectrometry of copurifying proteins, we previously identified 20 kinetochore proteins (KKT1–20) that localize to kinetochores in *T. brucei* (Akiyoshi and Gull, 2014; Nerusheva and Akiyoshi, 2016). These proteins have no obvious orthologues outside of kinetoplastids. More recently, KKT-interacting protein 1 (KKIP1) was identified as a kinetochore protein distantly related to Ndc80/Nuf2 based on similarity in the coiled-coil regions (D’Archivio and Wickstead, 2017). However, KKIP1 does not appear to have a CH domain, which is vital for the microtubule-binding activity of the Ndc80 complex. It is therefore unclear whether KKIP1 is a bona fide Ndc80/Nuf2–like protein. Affinity purification of KKIP1 from cross-linked cells led to the identification of six additional proteins (KKIP2–7) that localize to the kinetochore area during mitosis (D’Archivio and Wickstead, 2017), although it remains to be tested whether KKIP2–7 interact with kinetochore proteins. Very little is known about the function of these KKT and KKIP proteins.

The presence of putative DNA-binding motifs in KKT2 and KKT3 implies that these two proteins likely bind DNA (Akiyoshi and Gull, 2014). In contrast, none of the KKT or KKIP proteins have significant sequence similarity to the microtubule-binding domains present in canonical kinetochore proteins or microtubule-associated proteins, such as the CH domain (Ndc80/Nuf2, EB1; Hayashi and Ikura, 2003; Wei et al., 2007; Ciferri et al., 2008), winged-helix domain (Ska1; Jeyaprakash et al., 2012; Schmidt et al., 2012), TOG domain (XMAP215; Slep, 2010; Al-Bassam and Chang, 2011), or spectrin fold (PRC1; Subramanian et al., 2010). It therefore remains unknown which of the kinetochore proteins in kinetoplastids might bind microtubules.

It has been proposed that kinetoplastids represent one of the earliest-branching eukaryotes based on a number of unique ultrastructural and molecular features (Cavalier-Smith, 2010, 2013; Akiyoshi, 2016). Understanding how the kinetoplastid kinetochore mediates interactions with dynamic microtubules could therefore provide important insights into the evolutionary origins of kinetochore proteins as well as fundamental principles of kinetochore–microtubule coupling in eukaryotes. Furthermore, because the molecular basis of microtubule interaction is likely distinct from other species, the kinetochores of kinetoplastids could be targeted to specifically kill parasites that cause devastating diseases. In this study, we report the identification of the first microtubule-binding kinetochore component in *T. brucei.*

## Results

### KKT4 localizes at spindle microtubules in addition to kinetochores

**Figure 1.**
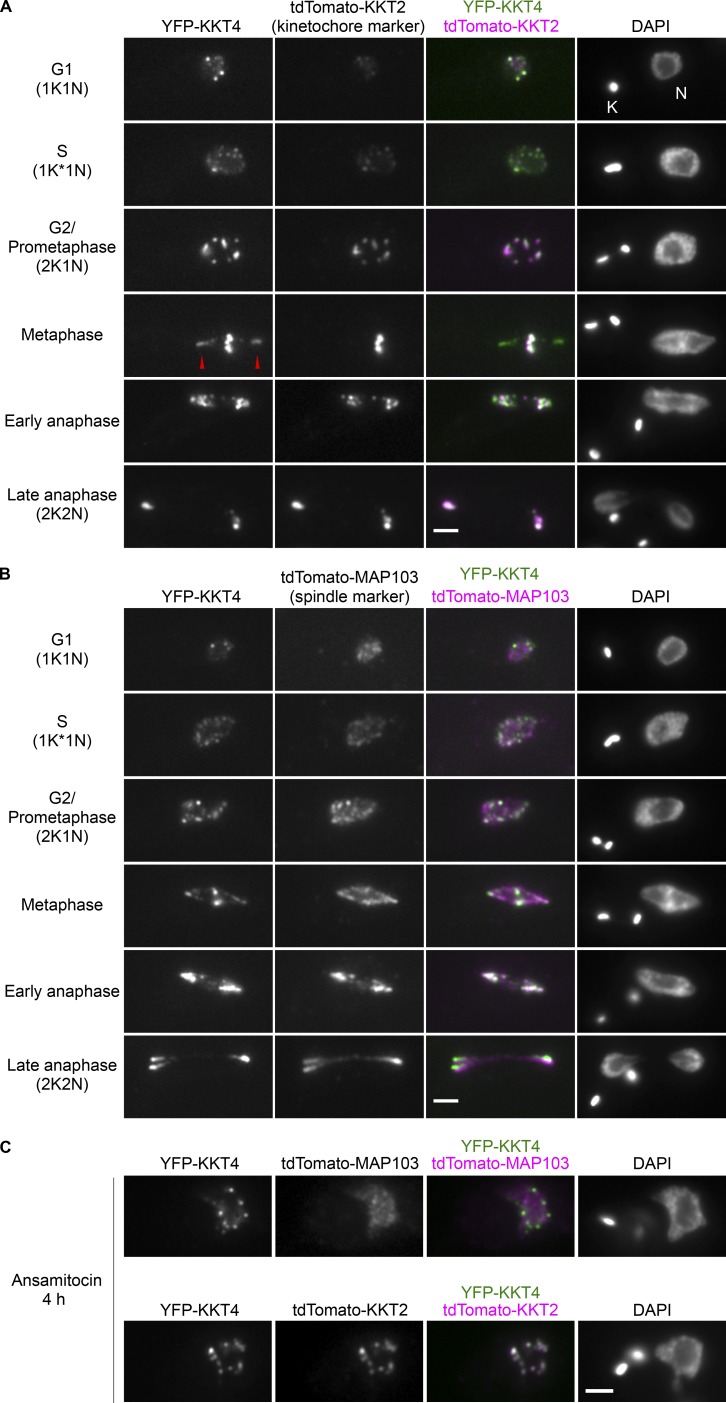
**KKT4 localizes at spindle microtubules in addition to kinetochores. (A)** Examples of cells expressing YFP-KKT4 and the kinetochore marker tdTomato-KKT2 at indicated cell-cycle stages (BAP665). Note that KKT4 has additional signals that do not colocalize with kinetochores, which are especially prominent in metaphase (arrowheads; *n* > 30 metaphase cells). K and N indicate the kinetoplast (mitochondrial DNA) and nucleus, respectively. These organelles have distinct replication and segregation timings and serve as good cell-cycle markers (Woodward and Gull, 1990; Siegel et al., 2008). K* denotes an elongated kinetoplast and indicates that the nucleus is in S phase. **(B)** Examples of cells expressing YFP-KKT4 and the spindle microtubule marker tdTomato-MAP103 showing that the nonkinetochore KKT4 signals partially colocalize with spindle microtubules (*n* > 20 metaphase cells; BAP943). **(C)** Examples of cells expressing YFP-KKT4 with tdTomato-MAP103 (top) or tdTomato-KKT2 (bottom) treated with 5 nM ansamitocin for 4 h and showing that KKT4 localizes at kinetochores even when bipolar spindle formation is perturbed (*n* > 20 cells in 2K1N). Bars, 2 µm.

In our previous analysis of endogenously YFP-tagged KKT proteins, we noticed that KKT4 localizes not only to kinetochores but also near the poles of the metaphase spindle (Akiyoshi and Gull, 2014). To further examine its cellular localization pattern, we used additional markers on kinetochores (KKT2) and on spindle microtubules (MAP103; Hayashi and Akiyoshi, 2018; Fig. 1, A and B). Imaging in different cell-cycle stages confirmed that KKT4 has nonkinetochore signal that colocalizes with a spindle marker, especially in metaphase cells. When cells were treated with a microtubule-destabilizing drug (ansamitocin), KKT4 localized solely at kinetochores (Fig. 1 C), as expected for a core kinetochore component. Interestingly, some microtubule-binding kinetochore components such as the Dam1 and Ska complexes also decorate spindle microtubules (Cheeseman et al., 2001; Hanisch et al., 2006). It is therefore conceivable that KKT4 associates closely with microtubules, possibly making direct contacts.

### KKT4 binds and diffuses along the microtubule lattice

**Figure 2.**
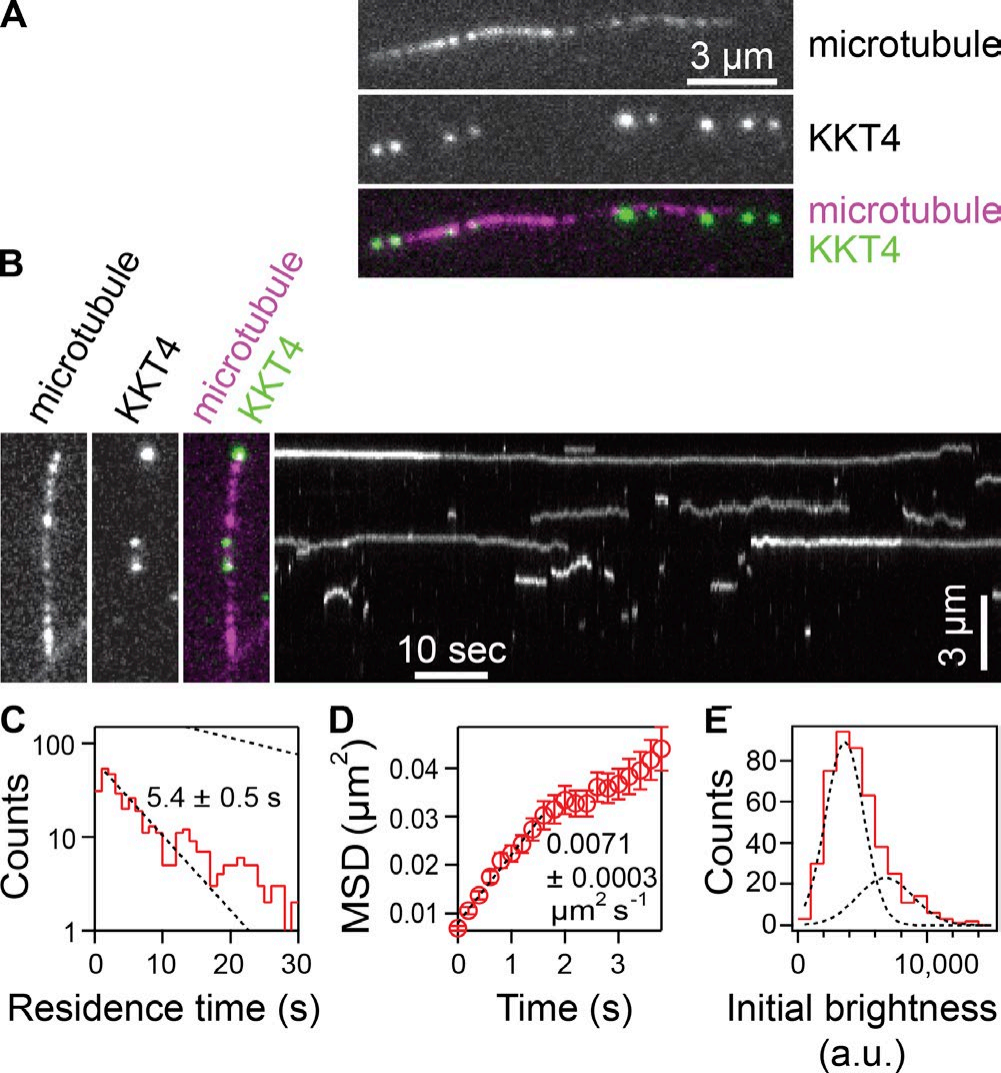
**KKT4 binds and diffuses on microtubules. (A)** Wild-type, fluorescent-tagged KKT4 particles (green) decorating a Taxol-stabilized microtubule (magenta). **(B)** Two-color fluorescence image (left) and corresponding kymograph (right) showing diffusion of the KKT4 particles on the microtubule lattice. **(C)** Distribution of residence times on microtubules for wild-type KKT4 particles. Lower dotted line shows exponential fit used to determine average residence time (*n* = 452 binding events on 48 microtubules). Upper dotted line shows exponential distribution of bleach times for single fluorescent-tagged KKT4 particles, corresponding to an average of *τ_bleach_* = 25 ± 1 s (*n* = 732 bleach events), which is long enough to ensure that KKT4 particles usually detached before bleaching. **(D)** Mean-squared displacement (MSD) of wild-type KKT4 particles plotted against time. Dotted line shows linear fit used to determine diffusion coefficient (*n* = 452 events). **(E)** Distribution of initial brightness values for wild-type KKT4 particles diffusing on Taxol-stabilized microtubules. Data are fitted by the sum of two Gaussians (dashed black curves) corresponding to a large population (72%) with a unitary brightness of 3,140 ± 1,470 a.u. and a small population (28%) with twice the brightness (mean ± SD; *n* = 452 particles).

To test whether KKT4 has microtubule-binding activity, we purified the full-length KKT4 protein fused with an N-terminal fluorescence and affinity tag, SNAP-6HIS-3FLAG. The protein was labeled during purification with a ^549^SNAP dye and eluted from beads using FLAG peptides (Fig. S1). We first tested whether the KKT4 protein binds Taxol-stabilized microtubules using total internal reflection fluorescence (TIRF) microscopy. Indeed, individual KKT4 particles bound transiently to coverslip-tethered microtubules, with an average residence time of 5.4 ± 0.5 s, and they diffused along the filaments with a diffusion coefficient of 0.0071 ± 0.0003 µm^2^/s (Fig. 2, A–D). This lattice diffusion is reminiscent of the behavior of major microtubule-binding kinetochore proteins from other organisms, such as the human and yeast Ndc80 complexes (Powers et al., 2009), the Dam1 complex (Westermann et al., 2006), and the Ska complex (Schmidt et al., 2012).

Kinetochores of more commonly studied organisms contain arrays of microtubule-binding elements. The avidity of such arrays is believed to be crucial for maintaining persistent attachments to microtubule tips that are continuously assembling and disassembling (Hill, 1985; Joglekar et al., 2006; Powers et al., 2009; Alushin et al., 2010). To estimate the number of KKT4 molecules per microtubule-bound particle, we fit their brightness distribution by the sum of two Gaussian functions (Fig. 2 E), corresponding to a large population (72%) with a unitary brightness of 3,140 ± 1,470 a.u. and a small population (28%) with twice the brightness (6,280 ± 2,160 a.u.). The unitary brightness was similar to that of an individual ^549^SNAP fluorophore, quantified from photobleaching experiments (Fig. S2, A and B). These data suggest that, under the conditions of our assay, KKT4 binds the microtubule lattice primarily in monomeric form. While most particles disappeared in a single step, which probably represents detachment from the microtubule, a small fraction showed a stepwise loss of half their intensity while they remained attached to the filament (Fig. 2 B). This observation is consistent with photobleaching of one fluorophore in a particle carrying two, and it suggests that KKT4 might have a tendency to oligomerize. We conclude that individual KKT4 molecules can bind microtubules directly, exhibiting lattice diffusion and a possible tendency to oligomerize, all of which are properties shared by core microtubule-binding kinetochore elements found in other organisms.

### Identification of basic residues important for KKT4 microtubule-binding activity

**Figure 3.**
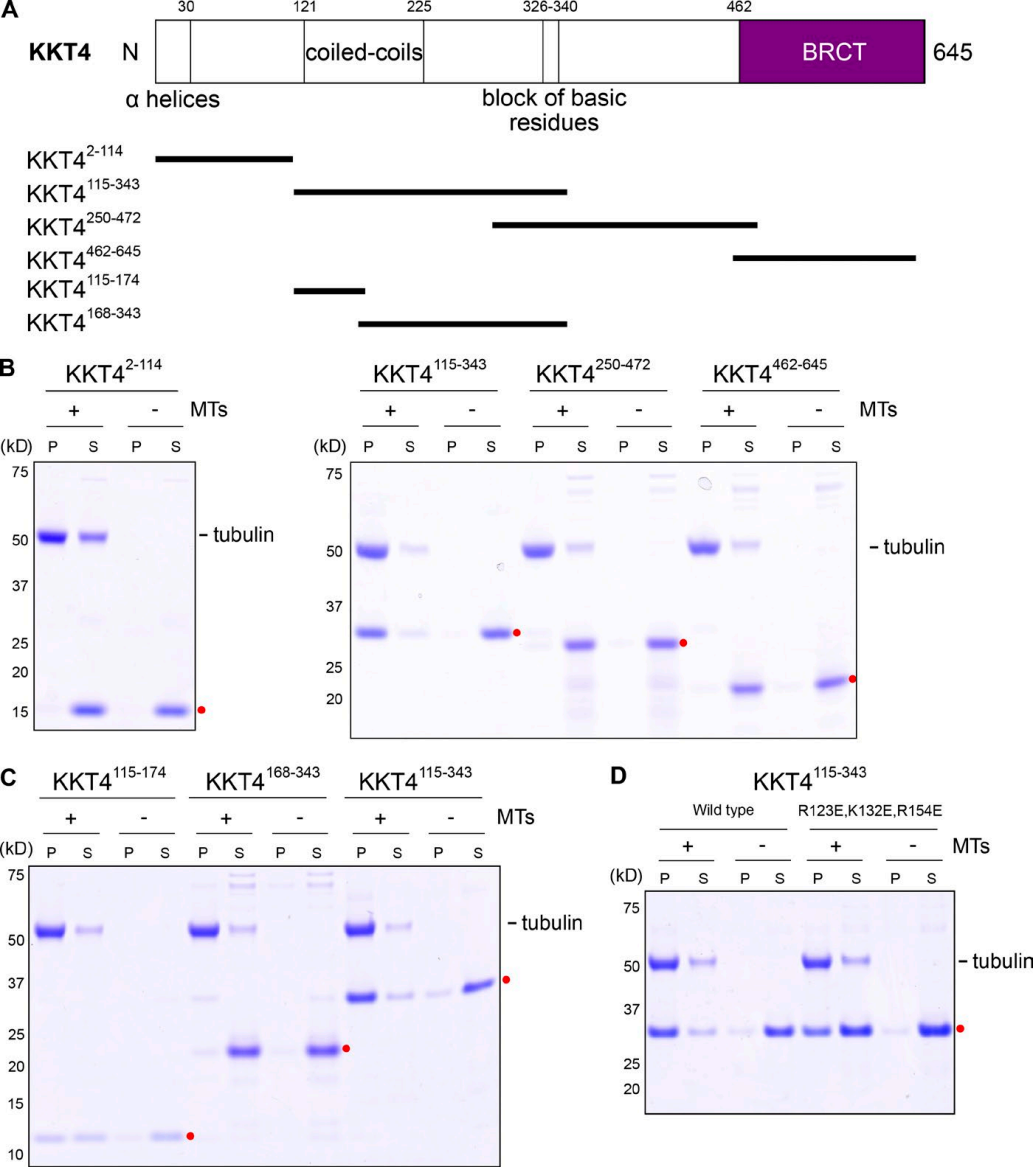
**KKT4^115–343^ is sufficient for microtubule binding. (A)** Schematic of the KKT4 protein sequence. Several features conserved among kinetoplastids are shown, with the truncated proteins used in Fig. 3 (B and C) indicated below. **(B)** Microtubule sedimentation assays of KKT4 fragments showing that KKT4^115–343^ can bind microtubules, whereas KKT4^2–114^, KKT4^250–472^, and KKT4^462–645^ do not. P and S stand for pellet and supernatant, respectively. Dots indicate KKT4 fragments tested in the assay. **(C)** Microtubule sedimentation assays of KKT4 fragments showing that KKT4^115–174^ can bind microtubules, albeit to a lesser extent than KKT4^115–343^. **(D)** The charge-reversal mutant KKT4 has reduced microtubule-binding activity.

We next aimed to define the microtubule-binding domain within KKT4. The protein has several features conserved among kinetoplastids, including N-terminal predicted α helices (residues 2–30), predicted coiled coils (121–225), a block of basic residues (326–340, predicted isoelectric point 11.0), and a C-terminal BRCA1 C terminus (BRCT) domain (462–645), which has been found in many DNA damage–response proteins and typically functions as a DNA- or phosphopeptide-binding domain (Reinhardt and Yaffe, 2013; Figs. 3 A and S3). We initially purified four truncated forms of KKT4 and tested them in microtubule cosedimentation assays. The central fragment with the predicted coiled coils and basic block (KKT4^115–343^) cosedimented robustly with Taxol-stabilized microtubules, but the other three constructs did not (Figs. 3 B and S4 A). We also found that corresponding protein fragments from *T. cruzi*, *T. congolense*, *Leishmania mexicana*, and *Phytomonas* cosedimented with microtubules (Fig. S4, B and C). KKT4 therefore has a microtubule-binding domain conserved among kinetoplastids.

**Figure 4.**
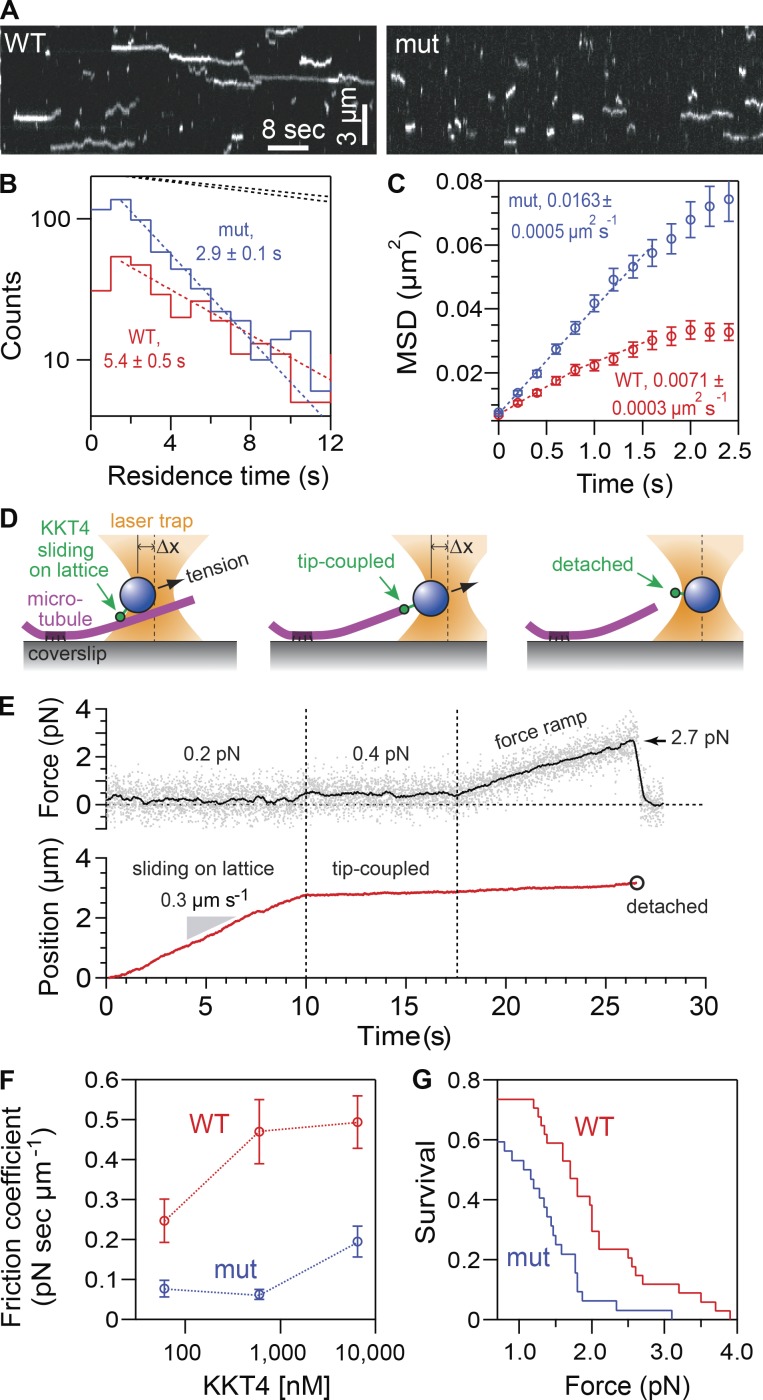
**Charge-reversal mutant KKT4 binds more weakly. (A)** Kymographs showing binding and diffusion of full-length, wild-type KKT4 and charge-reversal mutant KKT4 (mut) on Taxol-stabilized microtubules. **(B)** Distributions of residence times on microtubules for wild-type KKT4 (red) and charge-reversal mutant KKT4 (blue). Corresponding dotted lines show exponential fits used to determine average residence times (*n* > 452 binding events on >48 microtubules). Upper dotted lines show exponential bleach-time distributions for single wild-type and mutant KKT4 particles, corresponding to average bleach times of *τ_bleach_* = 25 ± 1 s and *τ_bleach_* = 30 ± 1 s, respectively. Wild-type data are recopied from Fig. 2 for comparison. **(C)** Mean-squared displacement (MSD) of wild-type KKT4 (red) and mutant KKT4 (blue) particles plotted against time. Dotted lines show linear fits used to determine diffusion coefficients (*n* > 452 particles). Wild-type data are recopied from Fig. 2 for comparison. **(D)** Schematic of laser trap assay used to measure friction coefficients and rupture strengths for KKT4^115–343^-decorated beads. **(E)** Example record showing trap force and bead displacement versus time. **(F)** Friction coefficients for wild-type KKT4^115–343^ (red) and charge-reversal mutant KKT4^115–343^ (blue) at indicated concentrations (mean ± SEM; *n* = 13–47 events). All individual friction coefficient values are given in Table S1. **(G)** Attachment survival probability versus force for wild-type KKT4^115–343^ (red) and charge-reversal mutant KKT4^115–343^ (blue; *n* = 33 and 35 events, respectively). All individual rupture force values are given in Table S1.

To further dissect the protein, we created two shorter constructs, KKT4^115–174^ and KKT4^168–343^. Of these, only KKT4^115–174^ cosedimented with microtubules (Fig. 3 C). However, we note that KKT4^115–174^ cosedimented less efficiently than KKT4^115–343^, implying that a feature within residues 175–343 enhances the microtubule-binding activity. Due to its positively charged residues, the predicted isoelectric point of KKT4^115–174^ is 9.8 (while that of KKT4^115–343^ is 9.7). Because microtubule binding is often mediated by electrostatic charges (Ciferri et al., 2008; Schmidt et al., 2012), we replaced three basic residues in the KKT4^115–343^ construct with acidic residues (R123E, K132E, and R154E). This charge-reversal mutant sedimented with Taxol-stabilized microtubules less efficiently than wild-type KKT4^115–343^ (Fig. 3 D), indicating that its microtubule-binding activity was partially compromised. Likewise, in TIRF experiments (Fig. 4 A), the average residence time on Taxol-stabilized microtubules for individual mutant KKT4 particles carrying the same set of charge-reversal mutations was approximately twofold shorter compared with wild-type KKT4 (Fig. 4 B), and their lattice diffusion was approximately twofold faster (Fig. 4 C). Similar changes in single-particle residence time and lattice diffusion occur when mutations or secondary modifications (phosphorylations) are introduced into the microtubule-binding domains of kinetochore proteins from other organisms (Gestaut et al., 2008; Umbreit et al., 2012; Zaytsev et al., 2014). Thus, our observations suggest that the positively charged residues R123, K132, and R154 make an important contribution to the microtubule-binding activity of KKT4.

### KKT4 forms load-bearing attachments to dynamic microtubule tips

Kinetochore–microtubule attachments in vivo must bear piconewton-scale loads, especially during metaphase, when bioriented chromosomes are subjected to tensile forces from opposing microtubules (Nicklas, 1988; Chacón et al., 2014; Ye et al., 2016). To measure the load-bearing capacity of KKT4-based attachments, we applied a computer-controlled laser trap assay developed previously for the study of kinetochore components from other organisms (Asbury et al., 2006; Powers et al., 2009; Franck et al., 2010). Polystyrene microbeads were decorated with purified KKT4^115–343^ protein and then attached to individual dynamic microtubules growing from coverslip-anchored seeds. Initially, the beads were attached laterally to the sides of the microtubules between the coverslip anchor and the growing filament tip (Fig. 4 D, left). This arrangement mimics the in vivo situation where kinetochores initially attach laterally to spindle microtubules (Hayden et al., 1990; Tanaka et al., 2005). Constant tensile forces of ∼1 pN, applied toward the plus end, caused the laterally attached beads to slide along the filament lattice, usually without detachment (Fig. 4 E), similar to the lateral sliding behavior seen previously in vitro with other kinetochore components (Asbury et al., 2006; Powers et al., 2009). Beads decorated with the charge-reversal mutant KKT4^115–343^ slid more easily along the microtubule than those decorated with the same concentrations of wild-type KKT4^115–343^. To quantify this difference, we calculated friction coefficients (Bormuth et al., 2009), dividing the applied force by the sliding speeds (Fig. 4 F and Table S1). The average friction coefficient for charge-reversal mutant-coated beads was substantially lower than that for wild type across a range of concentrations, indicating a weaker interaction of the mutant with the lattice.

Once a sliding bead reached the plus end of the microtubule, its movement abruptly slowed and was thereafter governed by the speed of microtubule assembly (Fig. 4 D, middle). This “tip-coupled” arrangement mimics the situation in vivo when kinetochores maintain load-bearing attachments to growing plus ends (Inoué and Salmon, 1995). To measure the strength of tip coupling, we increased the tension gradually (at 0.25 pN/s) until the attachment ruptured (Fig. 4 D, right). Tip attachments formed by wild-type KKT4^115–343^ ruptured on average at 2.1 ± 0.2 pN, a strength substantially less than couplers based on the yeast Ndc80 complex (generously provided by Jae ook Kim and Trisha Davis, University of Washington, Seattle, WA; Fig. S2, C and D), but nevertheless sufficient to contribute to the piconewton-scale forces expected at kinetochores in vivo. Attachments formed by the charge-reversal mutant KKT4^115–343^ were weaker than wild-type KKT4^115–343^, rupturing on average at 1.6 ± 0.1 pN (Fig. 4 G and Table S1). These observations establish that KKT4-based couplers can support significant loads and confirm that the strength of both lateral and end-on attachments to dynamic microtubules depends on the three basic residues (R123, K132, and R154).

### KKT4 tracks with dynamic microtubule tips and harnesses tip disassembly to produce force

**Figure 5.**
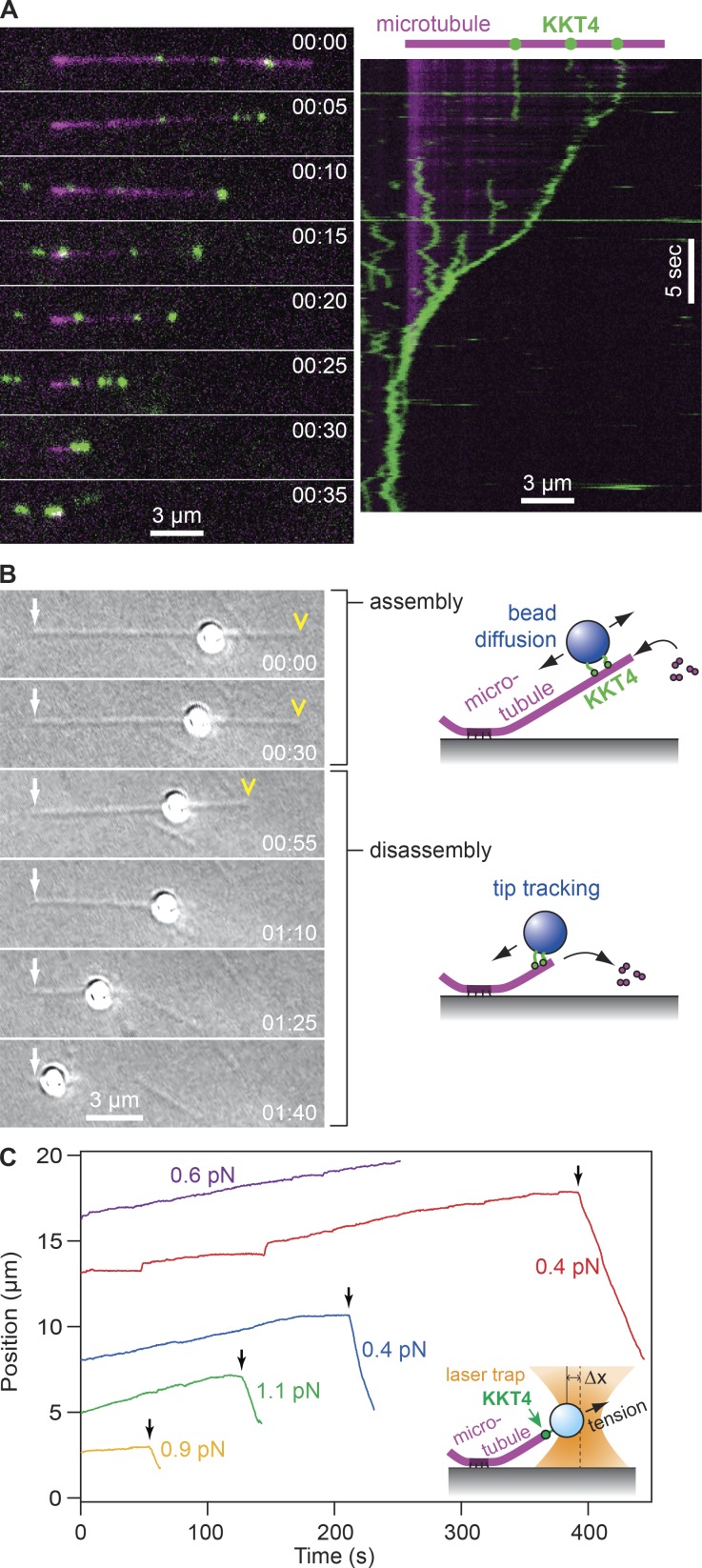
**KKT4 tracks with dynamic microtubule tips. (A)** Selected frames (left) and kymograph (right) from Video 1 showing wild-type KKT4 (green) tracking with the disassembling tip of a microtubule (magenta). Elapsed times are in minutes:seconds. Individual KKT4 particles can also be seen diffusing on the microtubule lattice. **(B)** Selected frames from Video 2 showing a wild-type KKT4^115–343^–coated bead diffusing on the microtubule lattice and then tracking with a disassembling tip. Arrows indicate the coverslip-anchored portion of the microtubule seed. Arrowheads indicate the microtubule tip. Elapsed times are in minutes:seconds. **(C)** Records of bead position versus time during continuous application of tensile force. Increasing position represents assembly-coupled movement in the direction of applied force, away from the coverslip-anchored seed (e.g., red trace, <400 s). Decreasing position represents disassembly-driven motion against the applied force (e.g., red trace, >400 s). Arrows indicate “catastrophe” events when the microtubule tip switched spontaneously from assembly into disassembly. For clarity, the records are offset vertically by an arbitrary amount. Inset: Schematic of laser trap assay. Statistical data for all recorded events are provided in Table S1.

The forces that move chromosomes during prometaphase, metaphase, and anaphase in vivo are generated in part via the tracking of kinetochores with dynamic microtubule tips (Inoué and Salmon, 1995). By maintaining a persistent, load-bearing attachment to a disassembling microtubule tip, a kinetochore harnesses energy released from the tip to produce mechanical work (Hill, 1985; Koshland et al., 1988; Coue et al., 1991). We found that purified KKT4 alone can recapitulate this tip-coupling activity. Using TIRF microscopy, we observed individual particles of fluorescently labeled full-length KKT4 bound along dynamic microtubules and then induced disassembly of the filaments by washing out free tubulin. When disassembling tips encountered KKT4 particles, the particles nearly always began tracking with the tips (26 of 28 particle-tip encounters [93%]) and were often carried all the way to the coverslip-anchored seed (Fig. 5 A and Video 1). Likewise, microbeads decorated with KKT4^115–343^ tracked persistently with disassembling microtubule tips in the absence of externally applied force (Fig. 5 B and Video 2; 11 of 13 beads tested [85%]). To test the load-bearing capacity, we applied continuous tension to tip-tracking beads using the laser trap. We found that KKT4^115–343^ reliably tracked with both assembling and disassembling microtubule tips under constant tensile forces of ∼1 pN (Fig. 5 C and Table S1). This load-bearing capacity is comparable to other microtubule-binding kinetochore components such as the Dam1 and Ndc80 complexes (Asbury et al., 2006; Powers et al., 2009).

### Depletion of KKT4 leads to a chromosome segregation defect

**Figure 6.**
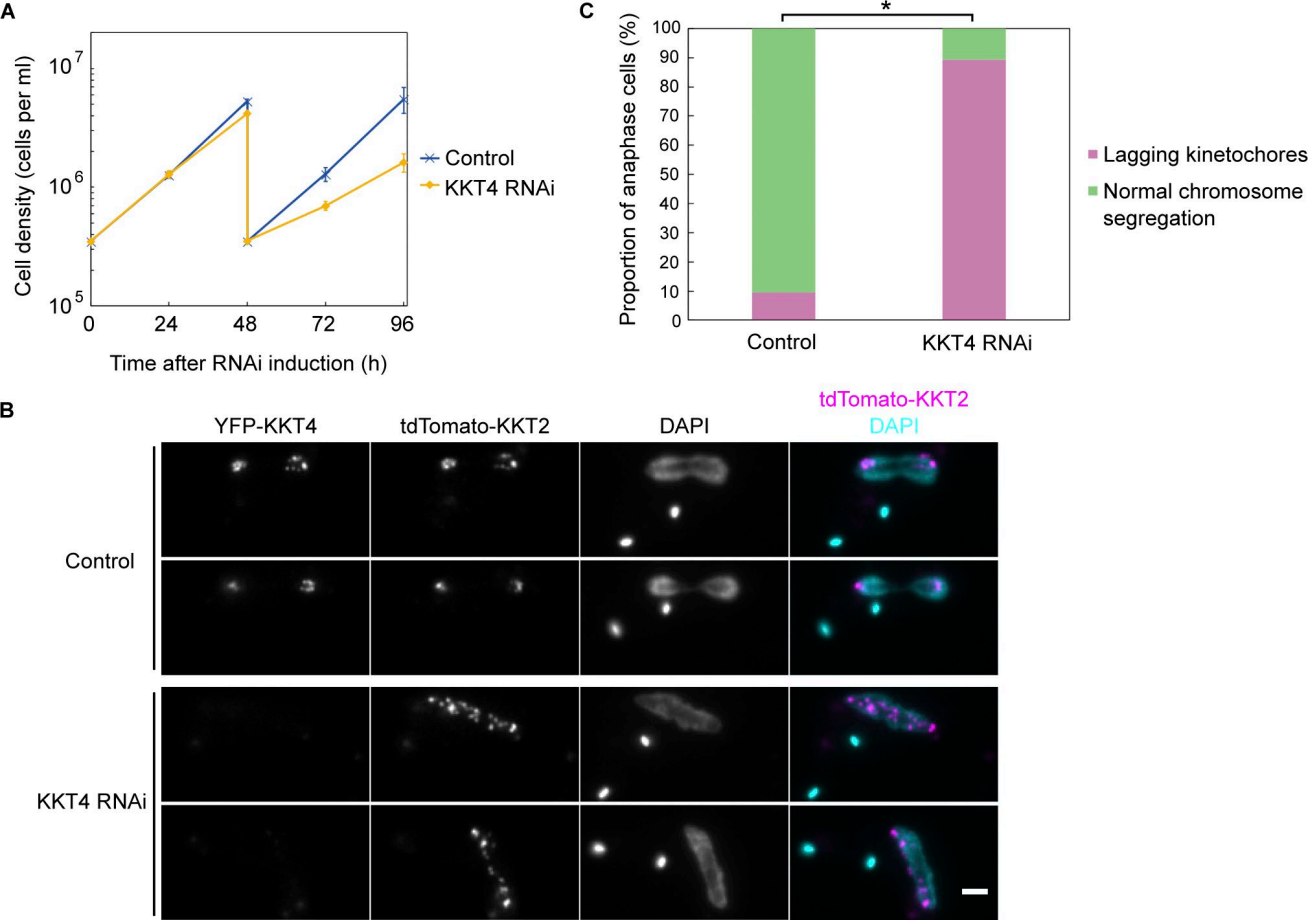
**KKT4 is essential for accurate chromosome segregation. (A)** RNAi-mediated knockdown of KKT4 leads to cell growth defects. Control is uninduced cell culture. Cultures were diluted at 48 h. Error bars represent SD from six experiments (BAP1082). Similar results were obtained using two independent RNAi constructs targeting different regions of the KKT4 transcript. **(B)** Cells expressing YFP-KKT4 and tdTomato-KKT2 were fixed at 48 h after induction, showing a number of lagging kinetochores in anaphase. Note that YFP-KKT4 signal was reduced by RNAi. Bar, 2 µm. **(C)** Quantification of anaphase cells with lagging kinetochores at 48 h postinduction showing that RNAi-induced cells have significantly more lagging kinetochores than uninduced control (*, P < 0.0001, Fisher’s exact test, *n* > 300 anaphase cells).

To test the importance of KKT4 for chromosome segregation in vivo, we performed RNAi-mediated knockdown in cells expressing YFP-tagged KKT4 together with another kinetochore marker (KKT2 tagged with tdTomato). The KKT4 signal was clearly reduced 48 h after RNAi induction, and growth retardation was observed after 72 h (Fig. 6, A and B). Chromosome segregation fidelity was examined after 48 h by monitoring kinetochore positions in anaphase cells. KKT4-depleted cells often had extensive lagging kinetochores (Fig. 6, B and C), showing that KKT4 is essential for proper chromosome segregation in vivo.

**Figure 7.**
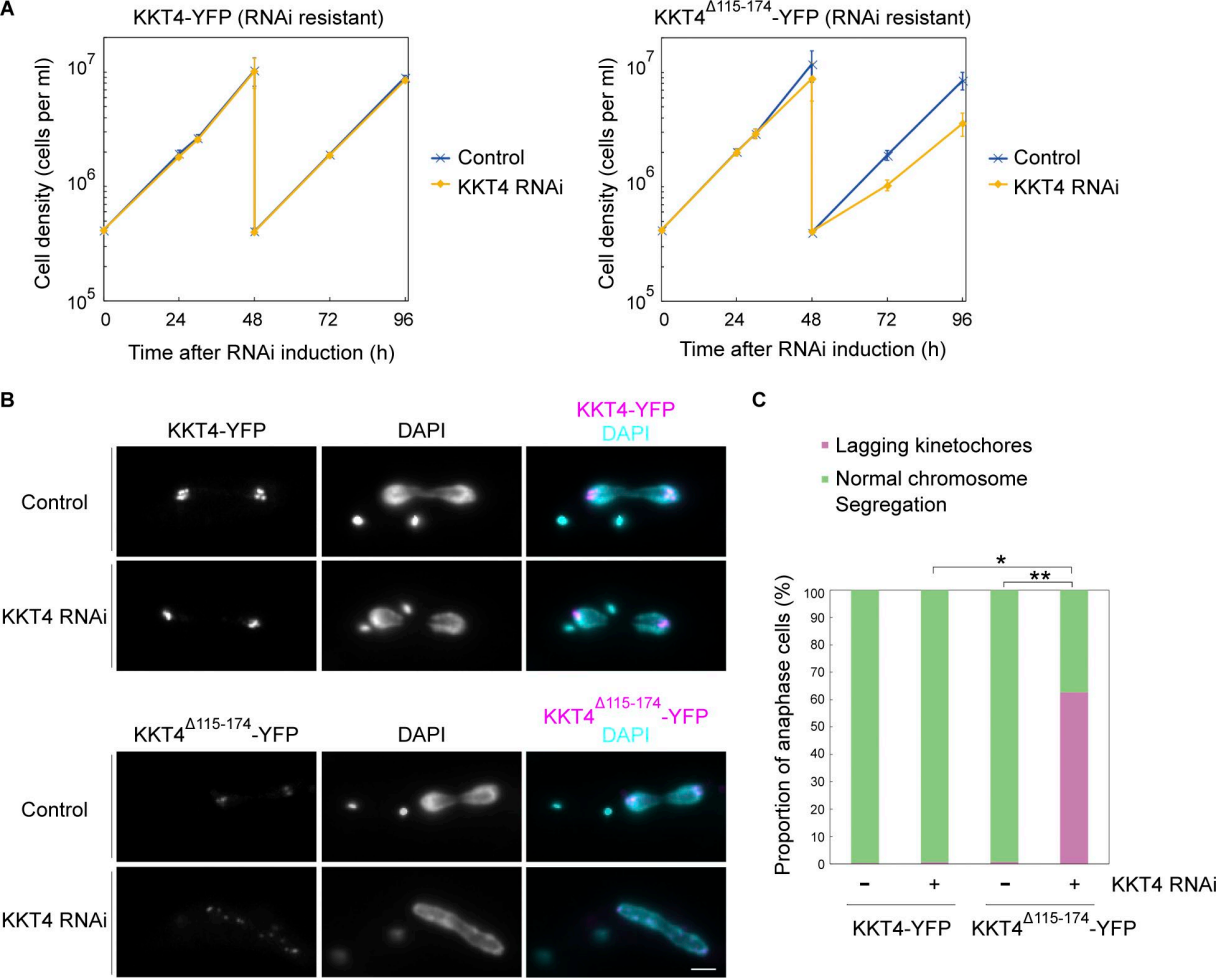
**KKT4^Δ115–174^ fails to support proper chromosome segregation. (A)** Expression of wild-type KKT4-YFP, but not KKT4^Δ115–174^-YFP, rescues the KKT4 3′ UTR–targeting RNAi phenotype. Controls are uninduced cell cultures. Error bars represent SD from three experiments (BAP1450 and BAP1484). **(B)** Examples of cells expressing KKT4-YFP (wild type or Δ115–174) fixed at 30 h postinduction and showing a number of lagging kinetochores in anaphase cells expressing KKT4^Δ115–174^. Bar, 2 µm. **(C)** Quantification of anaphase cells with lagging kinetochores at 30 h postinduction showing that RNAi-induced cells expressing KKT4^Δ115–174^ have significantly more lagging kinetochores than uninduced control (**, P < 0.0001, Fisher’s exact test) or RNAi-induced cells expressing wild-type KKT4 (*, P < 0.001, Fisher’s exact test; *n* > 300 anaphase cells for each).

We next tested the relevance of the identified microtubule-binding domain by expressing an RNAi-resistant form of KKT4 that has either the charge-reversal mutations (R123E, K132E, and R154E) or a deletion in the minimal microtubule-binding domain (residues 115–174). Both of these mutant proteins localized normally at kinetochores and near spindle poles in uninduced cells (Figs. 7 and S5 B). Upon RNAi induction, cells expressing the charge-reversal mutant grew normally, suggesting that the mutant retains sufficient microtubule-binding activity to support kinetochore–microtubule attachments in vivo (Fig. S5, A–C). In contrast, KKT4^Δ115–174^ failed to rescue the growth defect (Fig. 7), meaning that this mutant protein that lacks the microtubule-binding domain cannot support proper kinetochore–microtubule attachments. However, we noticed that the protein level of KKT4^Δ115–174^ was significantly lower compared with wild-type KKT4 (Fig. S5, D and E). We therefore cannot exclude the possibility that the observed phenotype is due to reduced protein level. Nevertheless, these results confirm the importance of KKT4 and its microtubule-binding domain for the proliferation of trypanosomes.

### KKT4 is an inner kinetochore protein

In many eukaryotes, microtubule-binding kinetochore components locate in the outer kinetochore, and their recruitment depends on the presence of DNA-binding inner kinetochore components (Cheeseman and Desai, 2008). Furthermore, DNA-binding kinetochore components often localize constitutively at centromeres, while microtubule-binding components typically localize only during mitosis. Although kinetochore assembly in *T. brucei* is cell-cycle regulated (Ogbadoyi et al., 2000; Akiyoshi and Gull, 2014), it remains unknown whether the kinetochores of kinetoplastids are organized hierarchically like those in other eukaryotes. The fact that KKT4 is found at kinetochores throughout the *T. brucei* cell cycle suggests that this microtubule-binding protein might locate closely to DNA (Akiyoshi and Gull, 2014).

**Figure 8.**
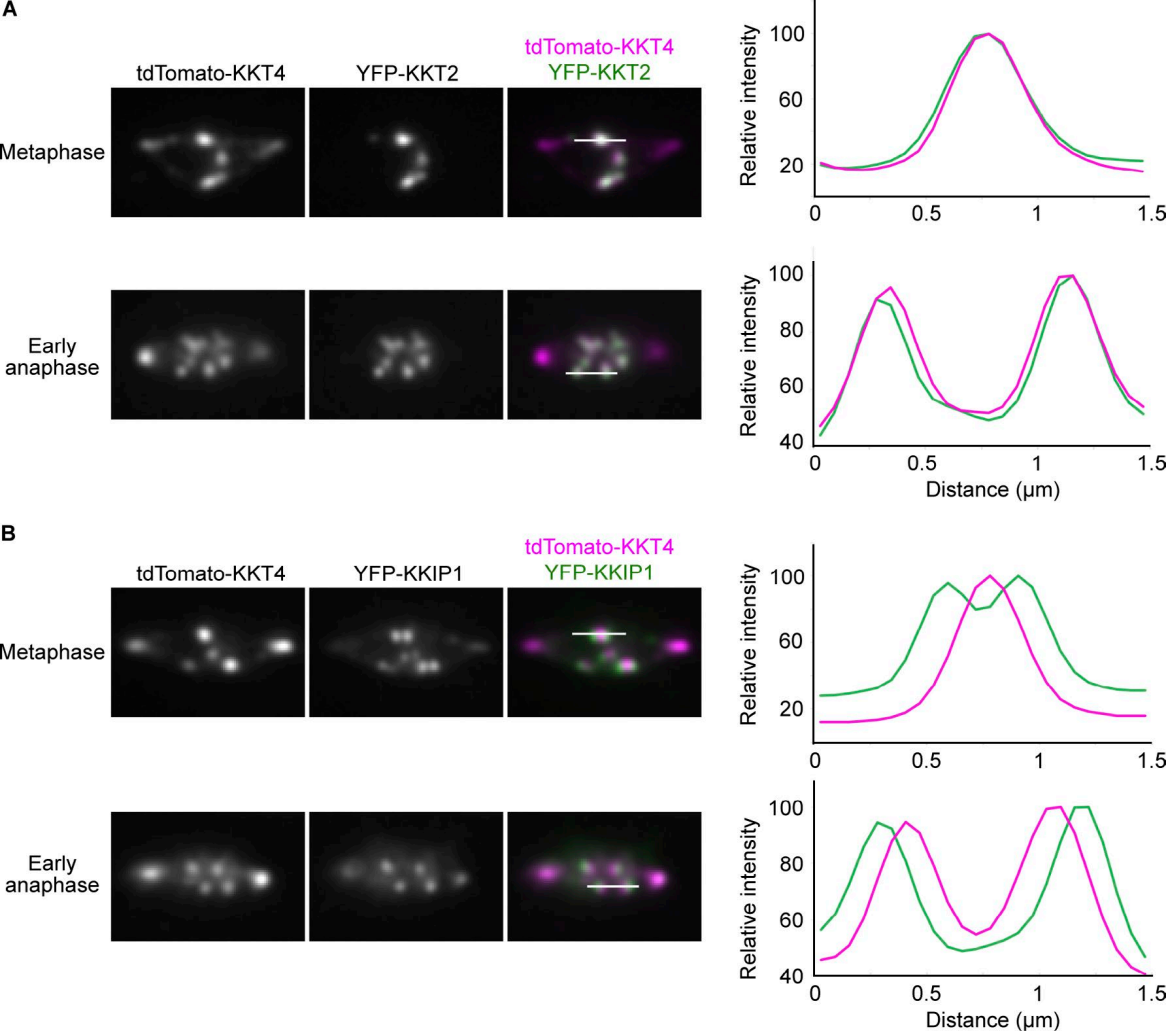
**KKT4 is an inner kinetochore protein. (A)** tdTomato-KKT4 colocalizes with YFP-KKT2 in metaphase (*n* > 25 cells) and early anaphase cells (*n* = 8 cells; BAP1272). Linescans for the area indicated by white lines (1.5 µm) are shown on the right. **(B)** YFP-KKIP1 appears as pairs of dots in metaphase cells, while tdTomato-KKT4 appears as individual dots (*n* = 25 cells). In early anaphase, KKIP1 is closer to spindle poles than KKT4 (*n* = 14 cells; BAP1273).

To map the arrangement of kinetochore components in *T. brucei*, we compared the location of KKT4 with that of the putative DNA-binding kinetochore protein KKT2 (Akiyoshi and Gull, 2014) as well as the outer kinetochore protein KKIP1 (D’Archivio and Wickstead, 2017). Sister kinetochores in all kinetoplastids studied thus far are held close together before anaphase, without the distinct separation between sister kinetochores that is commonly observed in other eukaryotes (Solari, 1980; Ureña, 1986; Ogbadoyi et al., 2000). Therefore, fluorescent-tagged KKT proteins in metaphase cells usually appear as individual dots rather than as pairs of dots (Akiyoshi and Gull, 2014). Linescans from two-color images confirmed that KKT4 colocalized closely with KKT2, with both proteins appearing as individual dots in metaphase cells and as separated sister pairs in anaphase cells (Fig. 8 A). In contrast, KKIP1 signals were already arranged in distinct pairs at metaphase, with individual KKT4 dots located between each pair of KKIP1 dots (Fig. 8 B, top). When sister kinetochores separated at early anaphase, KKIP1 remained closer to the spindle poles than KKT4 (Fig. 8 B, bottom). These observations reveal an unusual arrangement, with the microtubule-binding component KKT4 localizing to the inner kinetochore, together with the putative DNA-binding component, KKT2, rather than with the outer-kinetochore component, KKIP1.

**Figure 9.**
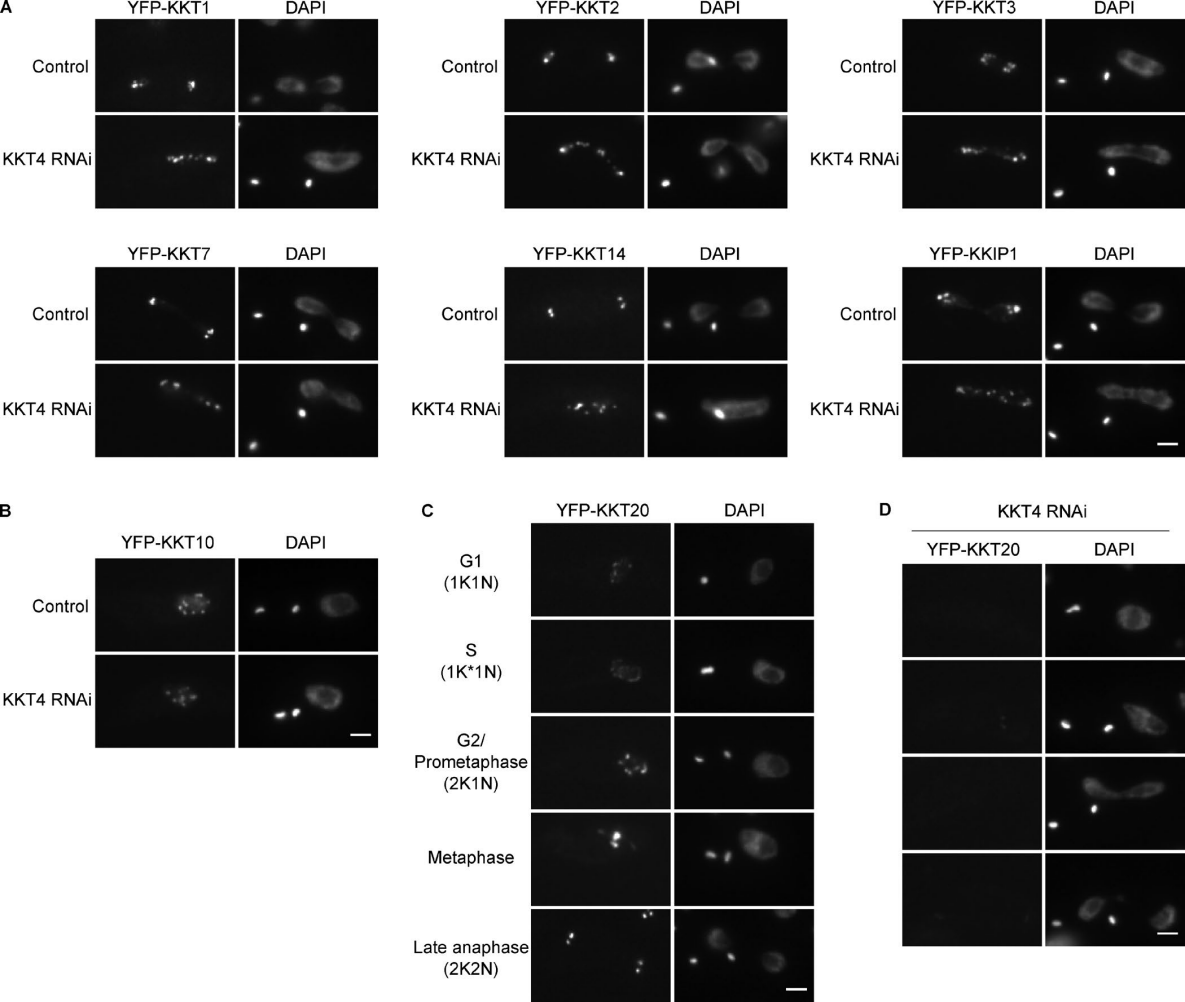
**KKT4 is dispensable for the localization of many kinetochore proteins. (A)** Kinetochore localization of YFP-tagged KKT1, KKT2, KKT3, KKT7, KKT14, and KKIP1 is not affected by KKT4 knockdown. Controls are uninduced cell cultures. Example of anaphase cells fixed 48 h postinduction are shown (*n* > 20 anaphase cells; BAP1236, BAP1237, BAP1238, BAP1240, BAP1242, and BAP1243). **(B)** Kinetochore localization of YFP-KKT10 is not disrupted by KKT4 knockdown (*n* > 20 cells in 2K1N; BAP1241). **(C)** The KKT20 short isoform (Tb927.8.4760.1:mRNA) localizes at kinetochores throughout the cell cycle in unperturbed cells (BAP1244). Note that the Tb927.8.4760.2:mRNA long isoform has less bright signal and localizes at kinetochores from S phase to anaphase (Nerusheva and Akiyoshi, 2016). **(D)** Kinetochore localization of the KKT20 short isoform is significantly diminished in >80% of cells upon KKT4 knockdown (*n* > 30 cells each in the 1K1N, 2K1N, and 2K2N category). Bars, 2 µm.

To determine whether KKT4 is required for the recruitment of other kinetochore components in *T. brucei*, we imaged eight different YFP-tagged kinetochore proteins in KKT4-depleted cells. Surprisingly, the localization of seven of them (KKT1, KKT2, KKT3, KKT7, KKT10, KKT14, and KKIP1) was unaffected by the depletion (Fig. 9, A and B). KKT4 was required only for the localization of KKT20 (Fig. 9, C and D). Thus, despite its location in the inner kinetochore, KKT4 is dispensable for the recruitment of most other kinetochore proteins. Taken together, the microtubule-binding activity of KKT4, its location in the inner kinetochore, and its dispensability for recruitment of other components raises a possibility that kinetochore architecture in kinetoplastids is distinct from that in other eukaryotes.

## Discussion

In this study, we report the identification of KKT4 as the first microtubule-binding kinetochore protein in *T. brucei*. Purified KKT4 alone tracks dynamic microtubule tips in vitro, albeit with lower strength than couplers based on the yeast Ndc80 complex. We speculate that additional microtubule-binding kinetochore proteins may exist in *T. brucei* that could strengthen KKT4-based coupling, similar to the accessory proteins that enhance Ndc80-based coupling in other eukaryotes (Welburn et al., 2009; Lampert et al., 2010; Tien et al., 2010; Miller et al., 2016; Helgeson et al., 2018). However, our preliminary experiments failed to detect significant microtubule-binding activities for KKT1, KKT6, KKT10, KKT13, KKT14, KKT16, KKT17, or KKT18. In the future, it will be important to reveal which proteins interact with KKT4 and to understand its regulatory mechanism. It is also possible that KKT4 has higher affinity for microtubules made from trypanosome tubulin than the bovine tubulin used in this study.

The microtubule-binding domain of KKT4 lacks significant sequence similarity to any other protein (Akiyoshi and Gull, 2014). Furthermore, no kinetochore protein known in other eukaryotes carries a BRCT domain (Musacchio and Desai, 2017). These observations suggest a unique evolutionary history for KKT4, starting from an ancestral BRCT-domain–containing protein. Lack of similarity to other microtubule-binding proteins also raises the possibility that KKT4 might interact with microtubules in a distinct manner. Structural analysis will be important for elucidating its mode of microtubule binding.

Unlike other microtubule-binding kinetochore proteins that localize to the outer area of kinetochores, KKT4 is an inner kinetochore protein. Interestingly, during the purification of full-length KKT4 protein from insect cells, we noticed copurification of insect DNA, which could be removed by heparin affinity chromatography. This observation suggests that KKT4 has DNA-binding activity in vitro. Localization of KKT4 constitutively to the inner kinetochore supports the possibility that KKT4 might also interact with DNA in vivo, which warrants further study. In prokaryotes, plasmid DNA segregation is mediated by a DNA-binding protein that also interacts with a cytoskeletal polymer (e.g., the ParR protein associates with *par*C DNA and the filament-forming ParM protein; Garner et al., 2007; Gerdes et al., 2010). We speculate that KKT4 could likewise bridge both DNA and microtubules. Furthermore, our previous finding that KKT4 copurifies with APC/C subunits (Akiyoshi and Gull, 2014) raises the possibility that KKT4 might directly regulate the timing of anaphase onset upon establishment of proper microtubule attachments. Further characterization of KKT4 will be vital for better understanding how the unconventional kinetochore of kinetoplastids performs conserved kinetochore functions.

## Materials and methods

### Trypanosome cells

All trypanosome cell lines used in this study were derived from *T. brucei* SmOxP927 procyclic form cells (TREU 927/4 expressing T7 RNA polymerase and the tetracycline repressor to allow inducible expression; Poon et al., 2012) and are listed in Table S2. Plasmids and primers used in this study are listed in Tables S3 and S4, respectively. Cells were grown at 28°C in SDM-79 medium supplemented with 10% (vol/vol) heat-inactivated fetal calf serum (Brun and Schönenberger, 1979). Cell growth was monitored using a CASY cell counter and analyzer system (Roche). Endogenous N-terminal YFP and tdTomato tagging was performed using the pEnT5-Y vector (Kelly et al., 2007) and pBA148 (Akiyoshi and Gull, 2014), respectively, using XbaI/BamHI sites. pBA215 (TY-tdTomato-MAP103–tagging construct) and pBA811 (TY-tdTomato-KKT4–tagging construct) were made by subcloning a XbaI/BamHI fragment of pBA31 (Hayashi and Akiyoshi, 2018) or pBA71 (Akiyoshi and Gull, 2014) into pBA148. For generation of an inducible KKT4 stem-loop RNAi construct (pBA1398), the following DNA fragment was synthesized by GeneArt and cloned into the pBA310 inducible expression vector (Akiyoshi and Gull, 2014), a HindIII site, a 428-bp fragment targeting KKT4 3′ UTR (starting at 63 bp downstream of the KKT4 stop codon), a stuffer sequence (5′-AAAGGCGGACCCTCATTTCTAAGTACGGTCAGGTGTCGTAGCACTGCATTGAATTCGATTGCCATTCTCCGAGTGTTTTAGCGTGACGGCCGCAGGGGTCCCATAA-3′), reversed orientation of the 428-bp fragment, and a BamHI site. Similar results were obtained using two independent RNAi constructs targeting the coding sequence of KKT4 (23–455 and 946–1,348; data not shown). Expression of RNAi-resistant forms of KKT4 was facilitated by endogenous C-terminal YFP-tagging constructs that were made from the pEnT5-Y vector (Kelly et al., 2007) using HindIII/SpeI sites (wild-type KKT4 [pBA1518], charge-reversal mutant [pBA1418], or KKT4^Δ115–174^ [pBA1610]). Plasmids linearized by NotI were transfected to trypanosomes by electroporation into an endogenous locus (pEnT5-Y and pBA148 derivatives) or 177-bp repeats on minichromosomes (pBA310 derivatives). Transfected cells were selected by the addition of 25 µg/ml hygromycin (pEnT5-Y derivatives), 10 µg/ml blasticidin (pBA148 derivatives), or 5 µg/ml phleomycin (pBA310 derivatives). At least three clones were analyzed for KKT4^Δ115–174^ and the charge-reversal mutant. RNAi was induced with doxycycline at a final concentration of 0.1 µg/ml. Ansamitocin P-3 was purchased from Abcam (ab144546), dissolved in DMSO to make 10 µM stock, and used at 5 nM final concentration. To examine the expression level of KKT4 proteins, two million cells were loaded onto SDS-PAGE gels, and immunoblots were performed by standard methods using anti-GFP mouse monoclonal antibodies at 1:200 (11814460001; Roche), anti-PFR2 mouse monoclonal antibodies at 1:1,500 (L8C4; Kohl et al., 1999), and anti-mouse IgG (HRP-conjugated, NA931; GE) at 1:5,000.

### Microscopy of trypanosome cells

Cells were fixed with formaldehyde as previously described (Nerusheva and Akiyoshi, 2016). Images were captured at room temperature on a DeltaVision fluorescence microscope (Applied Precision) installed with softWoRx version 5.5 housed in the Oxford Micron facility (https://www.micron.ox.ac.uk/home.php). Fluorescent images were captured with a CoolSNAP HQ camera using 60× objective lenses (1.42 NA) for lagging kinetochore quantifications (typically 16–21 z sections at 0.2- to 0.25-µm steps) or 100× objective lenses (1.4 NA) for all other experiments (12–20 z sections at 0.2- to 0.25-µm steps). Images were processed in ImageJ (Schneider et al., 2012). Figures were made using Inkscape (The Inkscape Team) or Illustrator (Adobe). Linescans were generated using the Plot Profile function in ImageJ (Schneider et al., 2012).

### Protein expression and purification from *Escherichia coli* and insect cells

DNA fragments encoding truncated KKT4 were amplified from genomic DNA or synthetic DNA (GeneArt) and cloned into the pNIC28-Bsa4 expression vector (gift of the Structural Genomics Consortium, University of Oxford, Oxford, UK) using a ligation-independent cloning method (Gileadi et al., 2008). *Leishmania mexicana* genomic DNA was kindly provided by Richard Wheeler, University of Oxford, Oxford, UK. Truncated KKT4 proteins fused with an N-terminal 6HIS tag and a TEV protease cleavage site were expressed in *E. coli* BL21(DE3) cells. Cells were grown in 2xTY media at 37°C to an OD_600_ of ∼0.8. Protein expression was induced by 0.1 mM IPTG and incubated overnight at 16°C. Cells were pelleted at 3,400 *g* at room temperature, and the cell pellet was frozen in liquid nitrogen and stored in −80°C. Cells were resuspended in lysis buffer (50 mM sodium phosphate, pH 7.5, 500 mM NaCl, and 10% glycerol) supplemented with protease inhibitors (20 µg/ml leupeptin, 20 µg/ml pepstatin, 20 µg/ml E-64, and 2 mM benzamidine) and 1 mM TCEP, and were sonicated on ice. Lysed cells were spun at 48,000 *g* at 4°C for 25 min. Supernatant was incubated with TALON beads (Takara Clontech) for 1 h at 4°C. We extensively washed the beads with lysis buffer and eluted proteins with elution buffer (50 mM sodium phosphate, pH 7.5, 500 mM NaCl, 10% glycerol, and 250 mM imidazole) with 1 mM TCEP. Buffer was exchanged into BRB80 (80 mM Pipes-KOH, pH 6.9, 1 mM EGTA, and 1 mM MgCl_2_) with 100 mM KCl using a PD MiniTrap G-25 column (GE). The sample was concentrated using a 3- or 10-kD MW Amicon concentrator (Millipore), and aliquots were flash-frozen in liquid nitrogen and stored at −80°C.

Synthetic DNA (GeneArt) encoding full-length KKT4 (codon-optimized for expression in insect cells) fused with an N-terminal SNAP-6HIS-3FLAG tag was cloned into the pACEBac2 vector (Geneva Biotech; Bieniossek et al., 2012). Bacmid was purified from DH10EmBacY *E. coli* cells using PureLink HiPure Plasmid Miniprep Kit (Thermo Fisher) and used to transfect Sf9 cells using Cellfectin II transfection reagent (Thermo Fisher). Sf9 cells were grown in Sf-900 II SFM media (Thermo Fisher). Baculovirus was amplified through three rounds of amplification. Typically, 500 ml of culture of Sf9 cells at 1–1.2 million cells/ml was infected with P3 baculovirus for ∼64 h before harvesting. Subsequent steps were performed at 4°C. Cells were pelleted at 700 *g* for 10 min, washed once with PBS, and resuspended in 10 ml BH0.25 (25 mM Hepes, pH 7.5, 2 mM MgCl_2_, 0.1 mM EDTA, 0.5 mM EGTA, 10% glycerol, and 250 mM NaCl) supplemented with 2× protease inhibitors (20 µg/ml leupeptin, 20 µg/ml pepstatin, 20 µg/ml E-64, and 0.4 mM PMSF) and 0.01 µg/ml DNase I. We added 0.2% NP-40 (IGEPAL CA-630) to the sample and lysed cells using a Dounce homogenizer (three rounds of 10 strokes with a 5-min break in-between). The sample was diluted by adding 12.5 ml BH0.25 with protease inhibitors and centrifuged for 30 min at 45,000 *g*. The supernatant was incubated with 2 ml anti-FLAG M2 affinity gel (Sigma) for 3 h with constant rotation, followed by five washes with BH0.25 with 1× protease inhibitors and 2 mM DTT (20 ml each). Beads were incubated with 2.5 ml of 20 µM ^549^SNAP dye (New England Biolabs) in BH0.25 with 1× protease inhibitors for 30 min at room temperature and subsequently washed twice with BH0.25 with 1× protease inhibitors and 2 mM DTT and twice with BH0.25 with 1× protease inhibitors. Proteins were eluted from the beads with gentle agitation of beads in 2 ml BH0.25 containing 0.5 mg/ml 3FLAG peptide (Sigma) and 1× protease inhibitors. The sample was further purified using 1 ml HiTrap Heparin HP column preequilibrated with 5% of buffer B (buffer A: 20 mM Hepes, pH 7.5, with 1 mM TCEP; buffer B: 20 mM Hepes, pH 7.5, and 1 M NaCl with 1 mM TCEP) and eluted with a linear gradient from 5% to 100% of buffer B. Fractions containing ^549^SNAP-tagged KKT4 were pooled (eluted at ∼340 mM NaCl), flash frozen in liquid nitrogen, and stored at −80°C before being used for TIRF or laser trap assays. Labeling was confirmed by an FLA 7000 scanner using the SHG532 laser and 0580 filter (GE). For analytical size-exclusion chromatography, the sample was concentrated to ∼24 µM by a 10-kD MW Amicon concentrator (Millipore) and loaded onto a Superose 6 increase 5/150 GL column (GE) equilibrated in gel filtration buffer (25 mM Hepes, pH 7.5, and 150 mM NaCl with 1 mM TCEP) on an ÄKTA pure 25 system. Protein concentration was determined by comparing the purified samples with BSA standards on SDS-PAGE gels as well as by protein assay (Bio-Rad).

Full-length *Saccharomyces cerevisiae* Ndc80 complex (6HIS tagged on the C terminus of the Spc24 protein) was expressed in *E. coli* using polycistronic vectors, affinity-purified using TALON resin (Clontech), and then purified further via gel filtration, as previously described (Wei et al., 2005; Powers et al., 2009).

### Cosedimentation assay

Taxol-stabilized microtubules were prepared as follows. We mixed 2.5 µl of 100 µM porcine tubulin resuspended in BRB80 with 1 mM GTP (Cytoskeleton), 1.25 µl BRB80, 0.5 µl of 40 mM MgCl_2_, 0.5 µl of 10 mM GTP, and 0.25 µl DMSO and incubated for 20 min at 37°C. Then, we added 120 µl BRB80 containing 12.5 µM Taxol (paclitaxel; Sigma) to the sample and passed it through a 27G 1/2-inch needle once to prepare sheared 2 µM microtubules. For microtubule cosedimentation assays, 20 µl KKT4 fragments (at final concentration of 4 µM) in BRB80 with 100 mM KCl and 20 µl microtubules (final, 1 µM) were mixed and incubated for 45 min at room temperature. For a no-microtubule control, we incubated KKT4 fragments with BRB80 with 12.5 µM Taxol. The samples were spun at 20,000 *g* at room temperature for 10 min, and the supernatant was taken. We added 40 µl of chilled BRB80 with 5 mM CaCl_2_ and put the samples on ice for 5 min to depolymerize microtubules. All samples were boiled for 3 min before analysis by SDS-PAGE gels stained with Coomassie brilliant blue R-250 (Bio-Rad). Cosedimentation assays were performed at least twice with similar results.

### TIRF-binding assays

Recombinant, ^549^SNAP-tagged, full-length, wild-type KKT4 or the charge-reversal mutant were used for all TIRF experiments. Flow channels were prepared using silanized coverslips as previously described (Gestaut et al., 2010; Kudalkar et al., 2016), coated with 1 mg/ml biotinylated BSA (Vector Laboratories), and thoroughly rinsed with BRB80. 1 mg/ml avidin DN (Vector Laboratories) was added, and the chamber was rinsed with BRB80. Biotinylated Taxol-stabilized microtubules (grown from bovine tubulin) were then introduced, allowed to bind the coverslip surface, and rinsed with a wash buffer of BRB80, 1 mM DTT, 10 µM Taxol, and 1 mg/ml κ-casein. Finally, 1.5 nM ^549^SNAP-tagged KKT4 was added in a buffer of BRB80, 1 mg/ml κ-casein, 1 mM DTT, 10 µM Taxol, 250 µg/ml glucose oxidase, 25 mM glucose, and 30 µg/ml catalase. The chamber was allowed to incubate for 5 min before imaging in a custom-built TIRF microscope (Deng and Asbury, 2017). Images were acquired at five frames per second for 100 s.

### TIRF depolymerization assays

Flow chambers were prepared and microtubules were attached using a method similar to that described above for Taxol-stabilized microtubule-binding assays, but with minor modifications. First, 1 mg/ml biotinylated BSA (Vector Laboratories) was flowed in and incubated for 5 min. The chamber was then rinsed with BRB80 followed by an incubation with 1 mg/ml avidin DN for 5 min. The chamber was again rinsed, and GMPCPP-stabilized microtubule seeds (Hyman et al., 1991; Asbury et al., 2006) were flowed in and allowed to bind the surface for 5 min. Excess seeds were removed by introducing microtubule growth buffer consisting of BRB80, 1 mM DTT, 1 mM GTP, and 1 mg/ml κ-casein. Then, in the same growth buffer, 10 µM free tubulin and 3.5 nM wild-type KKT4 were introduced. The chamber was incubated at 30°C for 10 min before imaging. Once it was clear that microtubule extensions of sufficient length had grown, their disassembly was promoted by exchanging buffer without tubulin but with 3.5 nM KKT4.

### Brightness and dwell time analysis

Custom TIRF analysis software was developed using LabView (National Instruments) as previously described (Gestaut et al., 2010; Asbury, 2016). This software generated kymographs of KKT4, which could be used to measure the position and brightness over time. Histograms of KKT4 particle brightness and dwell time on microtubules, and plots of mean squared displacement versus time, were generated using Igor Pro (WaveMetrics). The one-dimensional diffusion coefficients were calculated as *D* = <*x*^2^> (2*t*)^−1^ (Howard, 2001).

### Laser trap assays

Recombinant 6HIS-KKT4^115–343^ was attached to 0.56-µm-diameter streptavidin-coated polystyrene beads (Spherotech) using biotinylated His5 antibody (QIAGEN) as previously described (Franck et al., 2010). The amount of KKT4 per bead was adjusted by incubating dilutions of 60, 600, and 6,500 nM protein, prepared in BRB80, 8 mg/ml BSA, and 1 mM DTT with a fixed concentration of beads (0.025% wt/vol) at 4°C for 1 h. Our laser-trapping–based motility assay has been previously described (see, for example, Asbury et al., 2006; Powers et al., 2009; Franck et al., 2010). Briefly, dynamic microtubule extensions were grown from coverslip-anchored GMPCPP-stabilized microtubule seeds in a buffer consisting of BRB80, 1 mg/ml κ-casein, 1 mM GTP, 250 µg/ml glucose oxidase, 25 mM glucose, 30 µg/ml catalase, 1 mM DTT, and 6 µM (dimer) of purified bovine brain tubulin.

The laser trap has been previously described (Franck et al., 2010). Force feedback was implemented with custom LabView software. During clamping of the force, bead-trap separation was sampled at 40 kHz while stage position was updated at 50 Hz to maintain the desired load. Bead and stage position data were decimated to 200 Hz before storing to disk.

### Rupture force and friction coefficient measurements

Beads (at 0.025% wt/vol) were prepared with either wild-type or charge-reversal mutant KKT4^115–343^ at dilutions of 60, 600, and 6,500 nM. Individual beads were attached to the microtubule lattice and preloaded with a constant tension of 0.8 ± 0.3 pN. To measure the lateral friction coefficient (γ), we measured the force at which the bead was dragged along the lattice (F) and divided by the velocity at which the piezo stage moved (v), γ = F v^−1^. Once the bead reached the microtubule tip, the laser trap was programmed to ramp the force at a defined rate (0.25 pN/s) until the linkage ruptured. For experiments in which tip-tracking beads were observed, in the absence and presence of laser-trapping force (Fig. 5, B and C), the beads were coated with wild-type KKT4^115–343^ at 60 and 600 nM, respectively. For experiments with Ndc80 complex, the beads (at 0.025% wt/vol) were coated with 600 nM Ndc80 complex for comparison with the equivalent surface density of KKT4. Because Ndc80-coated beads exhibited more lateral friction than KKT4-coated beads, a higher initial preload tension of 3.3 ± 0.5 pN was required to drag the Ndc80-coated beads to the growing microtubule tips.

### Online supplemental material

Fig. S1 shows purification and characterization of full-length KKT4 used in TIRF assays. Fig. S2 presents photobleaching experiments (related to Fig. 2) as well as comparison between KKT4 and yeast Ndc80 complex in laser trap assays (related to Fig. 4). Fig. S3 is a multiple sequence alignment of KKT4 from various kinetoplastids. Fig. S4 shows that KKT4 proteins from four different kinetoplastids cosediment with microtubules. Fig. S5 shows that the charge-reversal mutant KKT4 supports chromosome segregation in vivo and that the protein level of the KKT4^Δ115–174^ mutant is lower than that of wild-type KKT4 (related to Fig. 7). Table S1 is a summary of optical trap–based bead motility assays (related to Figs. 4 (F and G), 5 C, and S2). Tables S2, S3, and S4 list trypanosome cell lines, plasmids, and primers and synthetic DNA sequences used in this study, respectively. Video 1 is a TIRF movie of wild-type KKT4 tracking with a disassembling microtubule tip (corresponding to Fig. 5 A). Video 2 shows that a bead decorated with wild-type KKT4^115–343^ diffuses on the microtubule lattice and then undergoes disassembly-driven motion (corresponding to Fig. 5 B).

## Supplementary Material

Supplemental Materials (PDF)

Table S1 (Excel)

Video 1

Video 2
