# Supplementary material for: The kinetoplastid kinetochore protein KKT4 is an unconventional microtubule tip–coupling protein
Source: J Cell Biol. 2018 Nov 5;217(11):3886–900. doi: 10.1083/jcb.201711181 (PMC6219724; doi:10.1083/jcb.201711181)
Supplement: Supplemental Materials (PDF) [file JCB_201711181_sm.pdf]

Supplemental material

Llauró et al., https://doi.org/10.1083/jcb.201711181

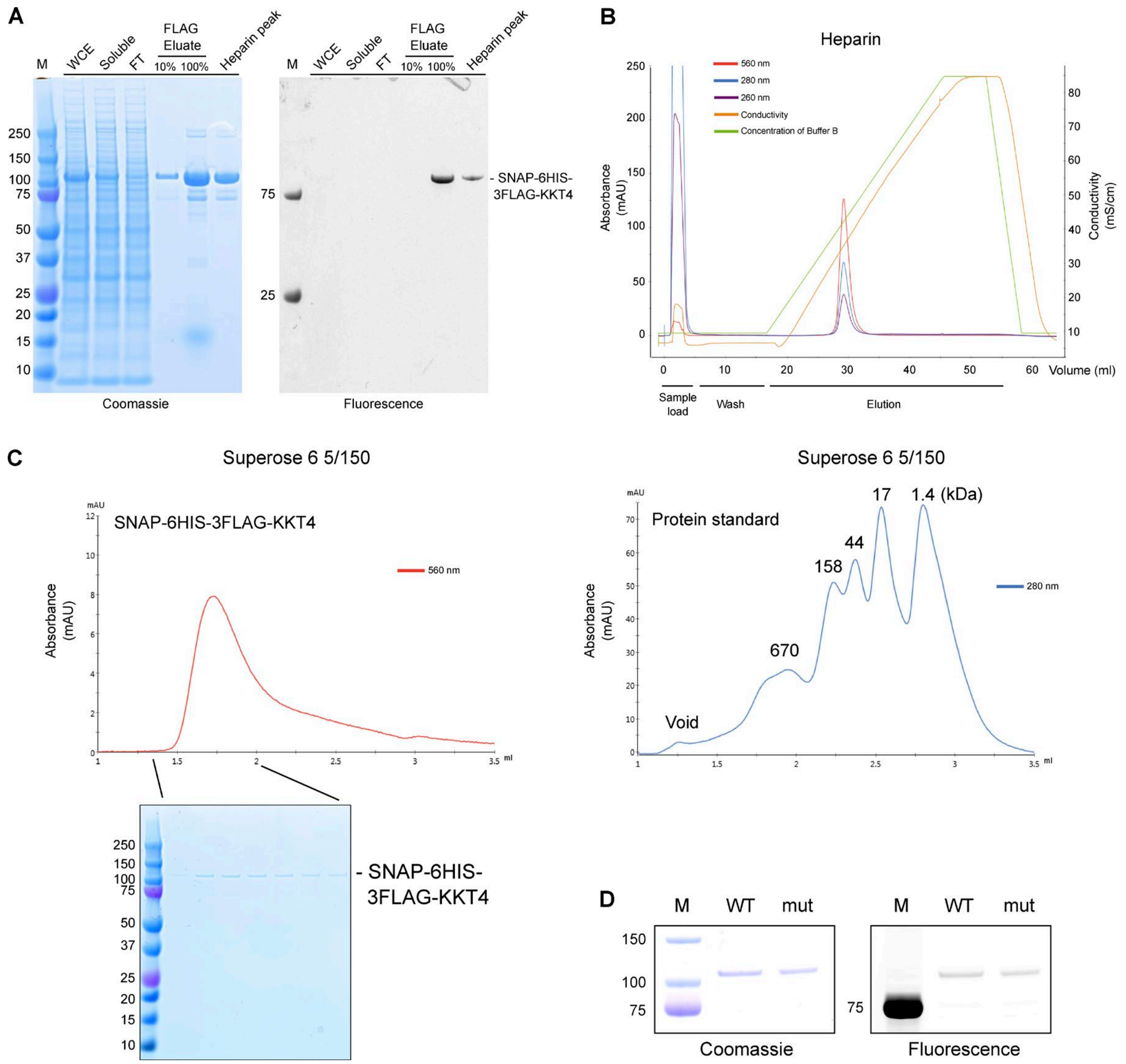

Figure S1. **Purification and characterization of full-length KKT4 used for TIRF assays. (A)** SNAP-6HIS-3FLAG-KKT4 was expressed and purified from insect cells. The protein was labeled with 549SNAP during purification. Left: Coomassie-stained SDS-PAGE gel. Right: Fluorescence scan of the same gel. FT, flow through; WCE, whole-cell extract. **(B)** Heparin chromatography of 549SNAP-6HIS-3FLAG-KKT4 sample to get rid of 3FLAG peptides and DNA contamination. **(C)** Size-exclusion chromatography of 549SNAP-6HIS-3FLAG-KKT4 showing that it migrates as a single peak in the Superose 6 5/150 column (left). Protein standard is shown on the right. **(D)** Purification of wild-type and charge-reversal mutant (mut; R123E, K132E, and R154E) of 549SNAP-6HIS-3FLAG-KKT4.

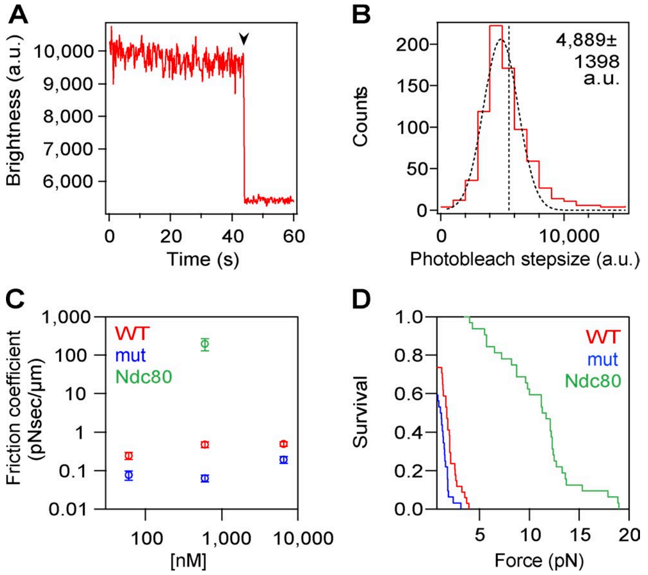

Figure S2. **Photobleaching experiments, and comparison between KKT4 and yeast Ndc80 complex. (A)** Record of brightness versus time for an individual wild-type, fluorescent-tagged KKT4 particle attached to a coverslip. The trace shows the stepwise loss of intensity corresponding to bleaching of a single fluorophore (arrowhead). **(B)** Distribution of photobleaching step sizes. Data are fitted by a Gaussian function (dashed black curve) corresponding to a population of single fluorophores with a modal brightness of 4,889 ± 1,398 a.u. The vertical dashed line corresponds to the mean brightness, 5,547 ± 2,478 a.u. (mean ± SD; n = 803 particles). **(C)** Friction coefficients for wild-type KKT4$^{115–343}$ (red), charge-reversal mutant KKT4$^{115–343}$ (mut; blue), and Ndc80 complex (green) at indicated concentrations (mean ± SEM; n = 13–47 events). Ndc80 complex was generously supplied by Jae ook Kim and Trisha Davis, University of Washington, Seattle, WA. **(D)** Attachment survival probability versus force for wild-type KKT4$^{115–343}$, charge-reversal mutant KKT4$^{115–343}$, and Ndc80 complex at 600 nM (n = 33–35 events). The values plotted for wild-type KKT4 and the charge-reversal mutant correspond to those given in Fig. 4 (F and G). All individual friction coefficients and rupture force values are given in Table S1.

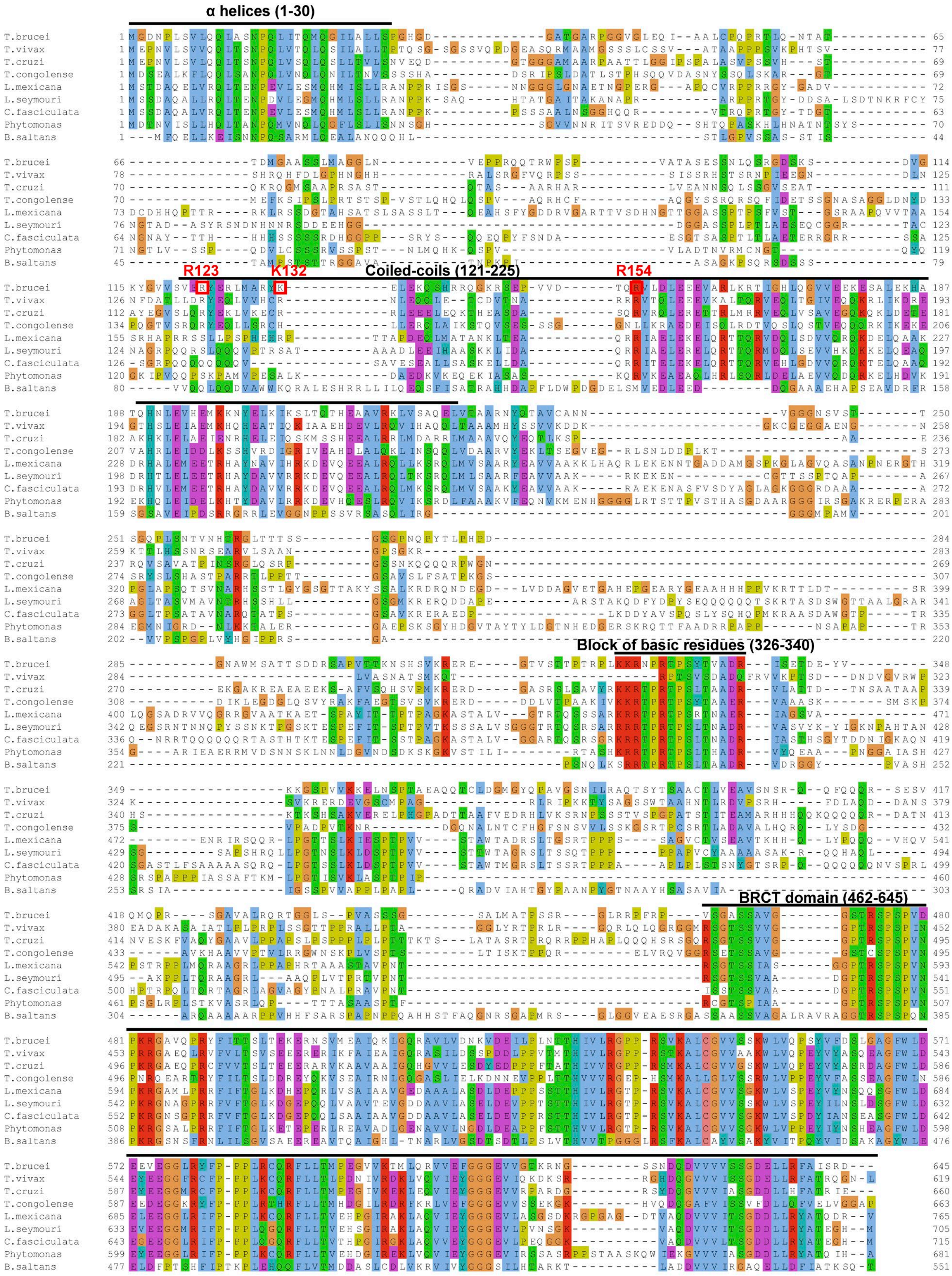

Figure S3. **Multiple sequence alignment of KKT4.** KKT4 proteins from various kinetoplastids were aligned using MAFFT (L-INS-i; Katoh and Standley, 2013) and visualized with CLUSTALX coloring scheme in Jalview (Waterhouse et al., 2009). Secondary structure predictions and coiled-coil predictions were performed using PSIPRED (Buchan et al., 2013) and COILS (Lupas et al., 1991), respectively. Charged residues mutated in this study are highlighted in red boxes.

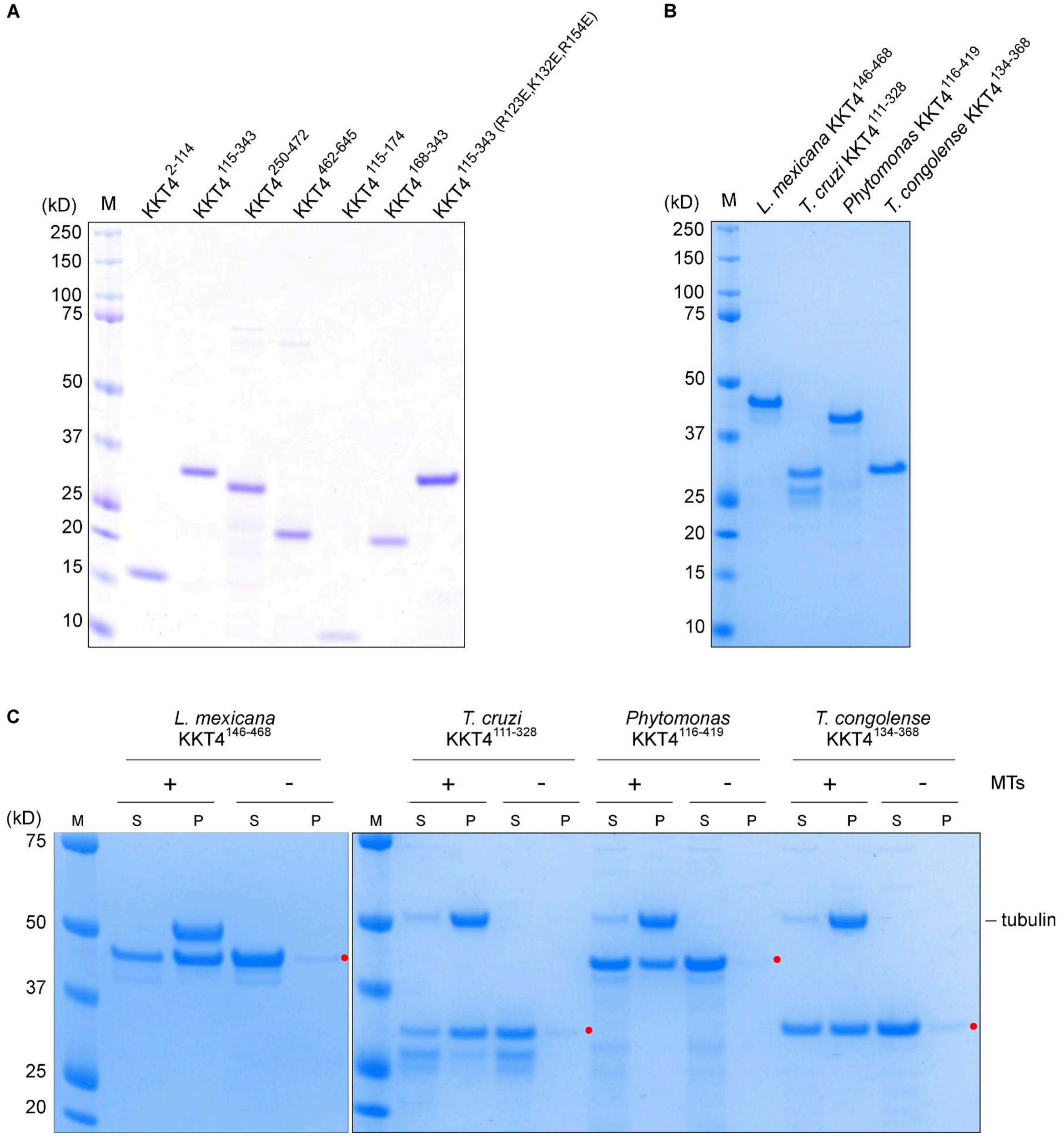

Figure S4. **KKT4 from four different kinetoplastids cosediment with microtubules. (A)** *T. brucei* KKT4 fragments fused with an N-terminal 6HIS tag were expressed and purified from *E. coli*. The samples were run on an SDS-PAGE gel and stained with Coomassie, showing the purity of each sample. **(B)** KKT4 fragments used in Fig. S4 C. **(C)** Microtubule (MT) sedimentation assays of KKT4 fragments from *L. mexicana*, *T. cruzi*, *Phytomonas*, and *T. congolense*.

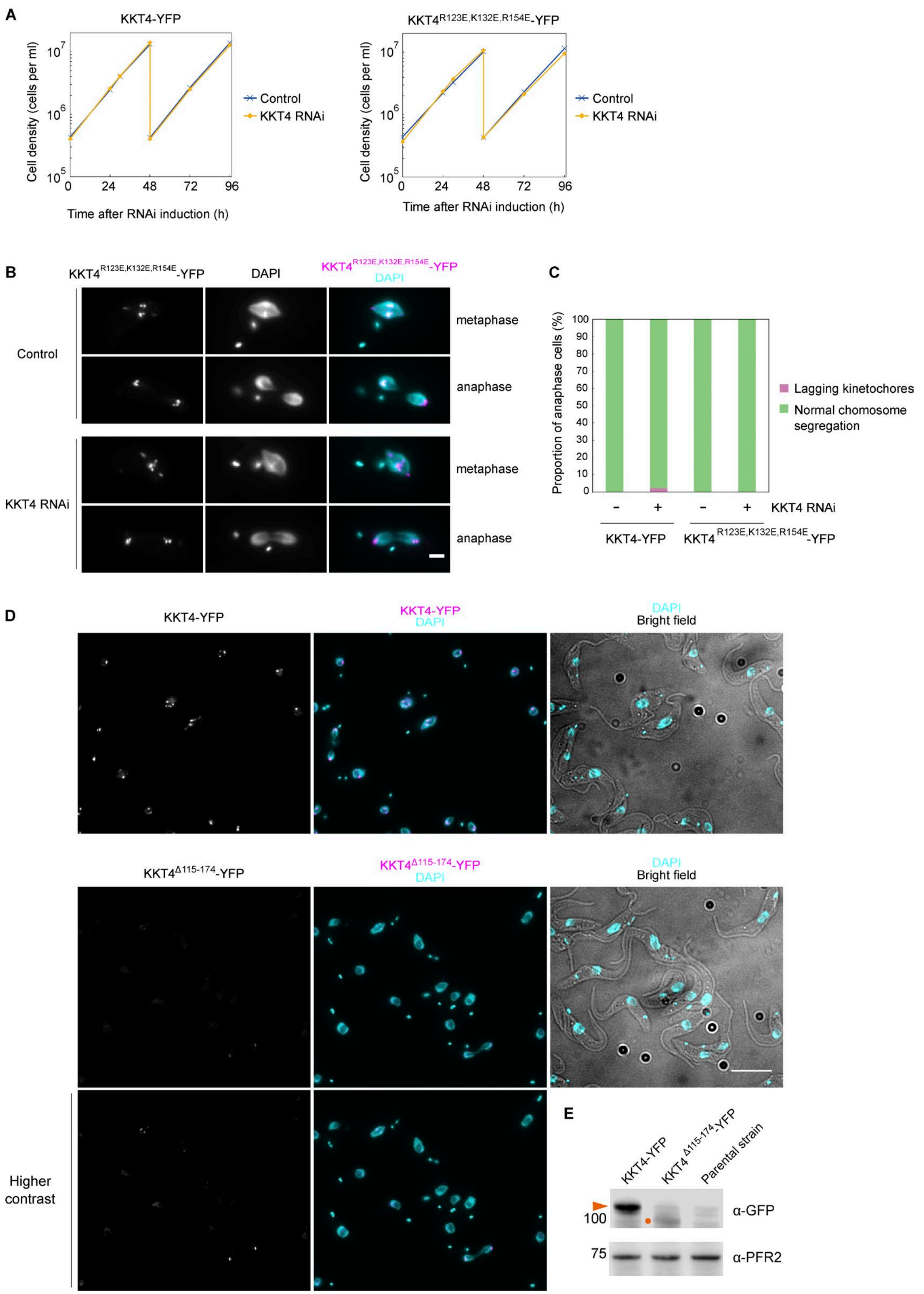

Figure S5. **Charge-reversal mutant KKT4 supports chromosome segregation in vivo. (A)** Expression of KKT4-YFP and KKT4^{R123E,K132E,R154E}-YFP rescues the KKT4 3′ UTR–targeting RNAi phenotype. Controls are uninduced cell cultures (BAP1450 and BAP1340). **(B)** The charge-reversal mutant localizes normally at kinetochores and near spindle poles. Cells were fixed at 30 h postinduction. Bar, 2 µm. **(C)** Quantification of anaphase cells with lagging kinetochores at 30 h postinduction ($n > 80$). **(D)** Examples of cells expressing KKT4-YFP (top) or KKT4$^{\Delta115-174}$-YFP (bottom) in uninduced cultures, showing that the KKT4$^{\Delta115-174}$-YFP signal is significantly lower than KKT4-YFP. Note that KKT4$^{\Delta115-174}$ localizes at kinetochores and near spindle poles in metaphase cells. Bar, 10 µm. **(E)** Immunoblots show reduced protein level for KKT4$^{\Delta115-174}$ (circle) compared with wild type (arrowhead). PFR2 was used as a loading control (BAP1450, BAP1484, and SmOxP9).

Table S2. **Trypanosome cell lines used in this study**

Strain	Description	Used in Figure
SmOxP9	Parental cell line that expresses TetR and T7 RNAP (Poon et al., 2012)	Fig. S5
BAP77	TY-YFP-KKT1 (Akiyoshi and Gull, 2014)	—
BAP122	TY-YFP-KKT2 (Akiyoshi and Gull, 2014)	—
BAP124	TY-YFP-KKT3 (Akiyoshi and Gull, 2014)	—
BAP126	TY-YFP-KKT7 (Akiyoshi and Gull, 2014)	—
BAP128	TY-YFP-KKT10 (Akiyoshi and Gull, 2014)	—
BAP163	TY-YFP-KKT14 (Akiyoshi and Gull, 2014)	—
BAP710	TY-YFP-KKIP1 (this study)	—
BAP831	TY-YFP-KKT20 (Tb927.8.4760.1:mRNA isoform; this study)	—
BAP1097	KKT4$^{R123E, K132E, R154E}$-YFP-TY (this study)	—
BAP1256	KKT4-YFP-TY (this study)	—
BAP1429	KKT4$^{\Delta 115–174}$-YFP-TY (this study)	—
BAP665	TY-YFP-KKT4, TY-tdTomato-KKT2 (this study)	Fig. 1
BAP943	TY-YFP-KKT4, TY-tdTomato-MAP103 (this study)	Fig. 1
BAP1082	TY-YFP-KKT4, TY-tdTomato-KKT2, KKT4 3′ UTR RNAi (this study)	Fig. 6
BAP1340	KKT4$^{R123E, K132E, R154E}$-YFP-TY, KKT4 3′ UTR RNAi (this study)	Fig. S5
BAP1450	KKT4-YFP-TY, KKT4 3′ UTR RNAi (this study)	Figs. 7 and S5
BAP1484	KKT4$^{\Delta 115–174}$-YFP-TY, KKT4 3′ UTR RNAi (this study)	Figs. 7 and S5
BAP1272	TY-YFP-KKT2, TY-tdTomato-KKT4 (this study)	Fig. 8
BAP1273	TY-YFP-KKIP1, TY-tdTomato-KKT4 (this study)	Fig. 8
BAP1236	TY-YFP-KKT1, KKT4 3′ UTR RNAi (this study)	Fig. 9
BAP1237	TY-YFP-KKT2, KKT4 3′ UTR RNAi (this study)	Fig. 9
BAP1238	TY-YFP-KKT3, KKT4 3′ UTR RNAi (this study)	Fig. 9
BAP1240	TY-YFP-KKT7, KKT4 3′ UTR RNAi (this study)	Fig. 9
BAP1241	TY-YFP-KKT10, KKT4 3′ UTR RNAi (this study)	Fig. 9
BAP1242	TY-YFP-KKT14, KKT4 3′ UTR RNAi (this study)	Fig. 9
BAP1243	TY-YFP-KKIP1, KKT4 3′ UTR RNAi (this study)	Fig. 9
BAP1244	TY-YFP-KKT20 (Tb927.8.4760.1:mRNA isoform), KKT4 3′ UTR RNAi (this study)	Fig. 9

Table S3. **Plasmids used in this study**

Name	Description
pEnT5-Y	TY-YFP tagging vector, hygromycin (Kelly et al., 2007)
pBA31	TY-YFP-MAP103 tagging construct, hygromycin (Hayashi and Akiyoshi, 2018)
pBA67	TY-YFP-KKT2 tagging construct, hygromycin (Akiyoshi and Gull, 2014)
pBA71	TY-YFP-KKT4 tagging construct, hygromycin (Akiyoshi and Gull, 2014)
pBA148	TY-tdTomato tagging vector, blasticidin (Akiyoshi and Gull, 2014)
pBA164	TY-tdTomato-KKT2 tagging construct, blasticidin (Nerusheva and Akiyoshi, 2016)
pBA1232	TY-YFP-KKT20 (Tb927.8.4760.1:mRNA isoform) tagging construct, hygromycin (this study)
pBA928	TY-YFP-KKIP1 tagging construct, hygromycin (this study)
pBA215	TY-tdTomato-MAP103 tagging construct, blasticidin (this study)
pBA811	TY-tdTomato-KKT4 tagging construct, blasticidin (this study)
pBA310	Inducible expression vector, integrate at 177 bp, phleomycin (Nerusheva and Akiyoshi, 2016)
pBA1398	Inducible KKT4 RNAi targeting the 3′ UTR, integrate at 177 bp, phleomycin (this study)
pBA472	Inducible GFP-NLS-KKT4$^{2–461}$, integrate at 177 bp, phleomycin (this study)
pBA1339	Inducible GFP-NLS-KKT4$^{2–461\ (R123E)}$, integrate at 177 bp, phleomycin (this study)
pBA1342	Inducible GFP-NLS-KKT4$^{2–461\ (R123E,\ K132E)}$, integrate at 177 bp, phleomycin (this study)
pBA1345	Inducible GFP-NLS-KKT4$^{2–461\ (R123E,\ K132E,\ R154E)}$, integrate at 177 bp, phleomycin (this study)
pBA838	Inducible GFP-NLS-KKT4$^{2–645}$, integrate at 177 bp, phleomycin (this study)
pBA1407	Inducible GFP-NLS-KKT4$^{2–645\ (R123E,\ K132E,\ R154E)}$, integrate at 177 bp, phleomycin, made by ligation-dependent subcloning using pBA1345 and pBA838 digested with PacI and Bsu36I (this study)
pBA1418	KKT4$^{R123E,\ K132E,\ R154E}$-YFP-TY tagging construct (1,735-bp homology region annealing to the 201–1,935 bp of KKT4 coding sequence), hygromycin (this study)
pBA1518	KKT4-YFP-TY tagging construct (1,735-bp homology region annealing to the 201–1,935 bp of KKT4 coding sequence), hygromycin (this study)
pBA1606	KKT4-YFP-TY tagging construct (1,935-bp homology region annealing to the 1–1,935 bp of KKT4 coding sequence), hygromycin (this study)
pBA1610	KKT4$^{\Delta 115-174}$-YFP-TY tagging construct (1,935-bp homology region annealing to the 1–1,935 bp of KKT4 coding sequence with deletion in 343–522 bp), hygromycin (this study)
pNIC28-Bsa4	pET expression vector with an N-terminal 6HIS tag, a TEV protease cleavage site, and a ligation-independent cloning site (Gileadi et al., 2008)
pBA1413	6HIS-KKT4$^{2–114}$ (this study)
pBA1065	6HIS-KKT4$^{115–343}$ (this study)
pBA1334	6HIS-KKT4$^{115–343\ (R123E,\ K132E,\ R154E)}$ (this study)
pBA775	6HIS-KKT4$^{250–472}$ (this study)
pBA569	6HIS-KKT4$^{462–645}$ (this study)
pBA1171	6HIS-KKT4$^{115–174}$ (this study)
pBA1417	6HIS-KKT4$^{168–343}$ (this study)
pBA1537	6HIS-*Lmexicana*KKT4$^{146–468}$ (this study)
pBA1753	6HIS-*Tcruzi*KKT4$^{111–328}$ (this study)
pBA1754	6HIS-*Phytomonas*KKT4$^{116–419}$ (this study)
pBA1755	6HIS-*Tcongolense*KKT4$^{134–368}$ (this study)
pBA925	SNAP-6HIS-3FLAG-KKT4 in pACEBac2 (this study)
pBA1351	SNAP-6HIS-3FLAG-KKT4$^{R123E,\ K132E,\ R154E}$ in pACEBac2 (this study)

Table S4. **Primers and synthetic DNA sequences used in this study**

To make	Primer or synthetic DNA sequences
pBA928	Following two PCR fragments were cloned into pEnT5-Y using XbaI and BamHI
	KKIP1 CDS targeting sequence with XbaI and NotI
	BA1452: GATCGATCTCTAGAGGAGCAGGTGAGTCTCCTGCGAAAGGTG
	BA1453: GATCGATCGCGGCCGCCTTCTGCTCAGCAACCAATG
	KKIP1 5′ UTR targeting sequence with NotI and BamHI
	BA1417: GATCGATCGCGGCCGCAAACCACAATGAGACTTTGTG
	BA1418: GATCGATCGGATCCCGACTGAAACAGAAACTACA
pBA1232	Synthetic DNA for the N-terminal tagging target sequence for KKT20 (Tb927.8.4760.1:mRNA isoform) with XbaI and BamHI, cloned into pEnT5-Y
	TCTAGAGGAGCAGGTAAGGACAAAAATAAGTGCACTTTTAGGTATTTTGAAGTGCTTCCAATAGATCATGAGCCCGAATACCAAAATGGCTAC<br>GTTTGTGATATTTGTTTTATGGAGTACTCCGAGGGTCCCTTTTTCCATTGCTCAAAGAGCGGAAAAGATGTGTGTCTGCGCTGTGGCGGTAAA<br>ATGGGGCTTACTCCTTTCACTGCACTGGTGAGCAAGGTAATGAGGCCTACTGTCTACTGGAGTGAGAGTGAGAGCTgcggccgcACGACAGGG<br>AAGCATTGGTTATGAAACTCAGTGTGCTTGTGTGTTCAATGTCGCGAACTACTGGGGTTGCATGGACATAGACATGTCTATTCATTTCTGTAT<br>GCATGTATACAGTCTCAGAAATTTCTCTACCACGGGCACGTGTATTTTCATTGTCTGAGTTTGAACTGCCACCACTAACCACTGAATGAGGTG<br>CGTATTATTTACACTTTATGTCATTACTTCTTCTTTTTTGACTATTGGAGGGACG GGATCC
pBA1398	KKT4 hairpin RNAi, 428-bp fragment starting from +63 ending at +490 bp of KKT4 3′ UTR with HindIII and BamHI, cloned into pBA310
	AAGCTTGATCAGCTGCTTTCCTCCACAAAGCGCTCCCAAAACCGAACCATGCGGGGCTCTGCGACACGAAAAGGATCGGTTATGTGCATTTCG<br>CTCACCTGACTATGGACGCCTCTGCGAATATATACGCGTGTAGTTTCATGAGGGTAGTGACTTTAGTGGGTGAAATGCACGGTTTAGGCGTGG<br>ATAAGGCCATTCACCGACGTTGTGTCAAATGTAGATGGTAAGAGGGTCTACACTGATAGAAGATATGATATTATCTTAATTGAAGGTGATTGT<br>TTGTTTACCTTATTTTTCGCTTAGTGAGGATTATGGGAAAATAATTATGATTTCTGCGCTTTCCTCTTGTTTTTGTTTTTGTCAGTCGACAAA<br>ACCGCACATACATATGAATGGTGATAGAAATGAAAGTTGATGTATGGCGTGACGTCTCAGAGAAAGGCGGACCCTCATTTCTAAGTACGGTCA<br>GGTGTCGTAGCACTGCATTGAATTCGATTGCCATTCTCCGAGTGTTTTAGCGTGACGGCCGCAGGGGTCCCATAACTCTGAGACGTCACGCCA<br>TACATCAACTTTCATTTCTATCACCATTCATATGTATGTGCGGTTTTGTCGACTGACAAAAACAAAAACAAGAGGAAAGCGCAGAAATCATAA<br>TTATTTTCCCATAATCCTCACTAAGCGAAAAATAAGGTAAACAAACAATCACCTTCAATTAAGATAATATCATATCTTCTATCAGTGTAGACCCTC<br>TTACCATCTACATTTGACACAACGTCGGTGAATGGCCTTATCCACGCCTAAACCGTGCATTTCACCCACTAAAGTCACTACCCTCATGAAACT<br>ACACGCGTATATATTCGCAGAGGCGTCCATAGTCAGGTGAGCGAAATGCACATAACCGATCCTTTTCGTGTCGCAGAGCCCCGCATGGTTCGG<br>TTTTGGG AGCGCTTTGTGGAGGAAAGCAGCTGATCGGATCC
pBA1413	To amplify KKT4$^{2–114}$ to insert into pNIC28-Bsa4
	BA1132: TACTTCCAATCCATGGGAGACAATCCACTGTCTGT
	BA1722: TATCCACCTTTACTGTCACCCTACATCGCTTTTGCTGTCACCACGC
pBA1065	To amplify KKT4$^{115–343}$ to insert into pNIC28-Bsa4
	BA1472: TACTTCCAATCCATGAAATATGGCGTCGTTTCTGTTGAGCG
	BA1268: TATCCACCTTTACTGTCATTCGGAAATCCTGTCGGCTAC
pBA1334	Site-directed mutagenesis primers to make KKT4$^{115–343\ (R123E,\ K132E,\ R154E)}$ from pBA1065 (these primers were also used to make pBA1339, pBA1342, and pBA1345 from pBA472)
	R123E
	BA1600: GGCGTCGTTTCTGTTGAGGAGTATGAGCGGCTCATGGCTCGC
	BA1632: GAGCCATGAGCCGCTCATACTCCTCAACAGAAACGACGCCATATTTCATGG
	K132E
	BA1602: CGGCTCATGGCTCGCTACGAGGAACTAGAGAAGCAATCACACCGGAG
	BA1601: GGTGTGATTGCTTCTCTAGTTCCTCGTAGCGAGCCATGAGCCGCTCAT
	R154E
	BA1604: CCCGTCGTCGATACGCAGGAGGTCCTTGATCTTGAAGAGGAAGTGGCG
	BA1603: CCTCTTCAAGATCAAGGACCTCCTGCGTATCGACGACGGGTTCAC
pBA775	To amplify KKT4$^{250–472}$ to insert into pNIC28-Bsa4
	BA1266: TACTTCCAATCCATGACCTCAGGGCAACCGCTTTC
	BA1135: TATCCACCTTTACTGTCAGGACCCTCCAACAGCAGAGG

Table S4. **Primers and synthetic DNA sequences used in this study *(Continued)***

To make	Primer or synthetic DNA sequences
pBA569	To amplify $KKT4^{462–645}$ to insert into pNIC28-Bsa4
	BA991: TACTTCCAATCCATGGTGAGCGGTGCCTCCTCTGC
	BA992: TATCCACCTTTACTGTCAATCACGACTTATAGCGAAAC
pBA1171	To amplify $KKT4^{115–174}$ to insert into pNIC28-Bsa4
	BA1472: TACTTCCAATCCATGAAATATGGCGTCGTTTCTGTTGAGCG
	BA1631: TATCCACCTTTACTGTCACCCCTGTAGATGTCCTATCG
pBA1417	To amplify $KKT4^{168–343}$ to insert into pNIC28-Bsa4
	BA1677: TACTTCCAATCCATGACGATAGGACATCTACAGGGGGTTGTTGAGG
	BA1268: TATCCACCTTTACTGTCATTCGGAAATCCTGTCGGCTAC
pBA1537	To amplify *L. mexicana* $KKT4^{146–468}$ from genomic DNA and insert into pNIC28-Bsa4
	BA1998: TACTTCCAATCCATG GCAGCGCCTCAGGTGGTCAC
	BA2003: TATCCACCTTTACTGTCACGACGTTGTCCCAGGCAGGC
pBA1753	Synthetic DNA sequence of *T. cruzi* Sylvio X10 $KKT4^{111–328}$ and primer sequences used to clone into pNIC28-Bsa4 (codon optimized for expression in bacterial cells)
	ACCGCATATGAAGGTGTTAGCCTGCAGCGTTATGAAAAACTGGTTAAAGAATGTCGTCGCCTGGAAGAAGAACTGGAACAGAAAACCCATGAA<br>GCAAGTGATGCAAGCCAGCGTGTTCGTCAGCTGGAACGTGAAACCACACGTCTGATGCGTCGTGTTGAACAGCTGGTTAGCGCAGTTGAAGGT<br>CAGAAACAGAAACTGGATGAAACCGAAGCAAAACATAAACTTGAACTGGCCGAAATTGAAAACCGTCATGAACTGGAAATTCAGAGCAAAATG<br>AGCAGCCATGAAGAAGCACTGCGTCGTCTGATGGATGCACGTCGCCTGATGGCAGCAGCAGTTCAGTATGAACAGACCCTGAAAAGTCCGGCA<br>GCAGAACGTCAGGTTTCAGCAGTTGCAACCCCGATTAATAGCCGTGGTCTGCAGAGCCGTCCGGGTAGCAGCAATAAACAGCAGCAACAGCGT<br>CCGTGGGGTAATGAAAAAGGTGCAAAACGTGAAGCAGAAGCCGAAGAAAAAAGCGCATTTGTTAGCCAGCATAGCGTTCCGATGAAACGTGAA<br>CGTGATGGTGCAAGCCGTAGCCTGAGCGCAGTTTATCGTAAAAAACGTACACCGCGTACGCCGAGTCTGACCGCAGCAGATCGTGTTCTGGCA<br>ACC
	BA2244: TACTTCCAATCCATGACCGCATATGAAGGTGTTAG
	BA2247: TATCCACCTTTACTGTCAGGTTGCCAGAACACGATCTG
pBA1754	Synthetic DNA sequence of *Phytomonas* $KKT4^{116–419}$ and primer sequences used to clone into pNIC28-Bsa4 (codon optimized for expression in bacterial cells)
	ACCCAGTATCAGGGTAAAATTCCGGTTCAGCAGCCGAGCAAACCGGCAATGGTTCCGGAAAGCGCACTGAAAGATGCAGAAGATAAAGTCAAA<br>GAGCAAGAGAAAATTGCAAGCGCAAGCAAACAGCGTGTTAAAGAAGCAGAAGCACAGCTGCATCGTCTGAGCCAGCGTCTGGATGAACTG<br>GCAGAAGTTGTTCAGGATCAGCGTAAAGAACTGCATGATGTGAAAGAAAAACACCAGCTGGAAATCGATGAACTGAAACATACCTATGATGCC<br>GTTCTGCGTCGTAAAGATGAAGTTCATCAAGAAAGCCTGCGTCAGGTGATTAAAAGCCGTGACCTGTTTGCAGCAGCAAAAGTTTTTGAACAG<br>AACGTGAAAATGGAAAATCATGGTGGTGGTGGCCTGCGTACCAGCACCACACCGGTTAGCACCCATGCAAGCGGTGATGCAGCACGCGGTGGT<br>GGTATTCGTAGCGGTGCAAAACGTGAACGTCCGGAACGTGCAGAAGGTATGAATATTGGTCGTGACAATCTGAAAAAAACCGCACTGGAACGT<br>GGTGCAGAACCGAGCAAAAGCGGTTATCATGATGGTGTTACCGCATATACCTATCTGGATGGCACCAATCATGAAGATGGTGAACGTAGCAAA<br>CGTCAGACCACCTTTGCAGCCGATCGTCGTCCGGCACCGCCTAATAGTGCACCGGCACCGACACGTGGTGCCCGTATTGAAGCCGAACGTCGT<br>ATGGTTGATAGCAATAATAGCAAACTGAATAACCTGGATGGCGTGAACGATTCCGATAAAAGCAAAGGTAAAGTTAGCACCATTCTGATTCGT<br>ACCGCAAGCCATAAACGTCGTACACCGCGTACGCCGAGTCTGACCCATGCAGATCGTGTTTATCAAGAAGCAGCACCG
	BA2245: TACTTCCAATCCATGACCCAGTATCAGGGTAAAAT
	BA2248: TATCCACCTTTACTGTCACGGTGCTGCTTCTTGATAAA
pBA1755	Synthetic DNA sequence of *T. congolense* $KKT4^{134–368}$ and primer sequences used to clone into pNIC28-Bsa4 (codon optimized for expression in bacterial cells)
	CCGCAGGGTACAGTTAGCCGTCAGCGTTATGAACAGCTGCTGAGCCGTTGTCATCTGCTGGAACGTCAGCTGGCAATTAAAAGCACCCAGGTT<br>AGCGAAAGCAGCAGCGGTGGTAATCTGCTGAAACGCGCAGAAGATGAAATTAGCCAGCTGCGTGATACCGTTCAGAGCCTGCAGAGCACCGTT<br>GAACAGCAGCAACGTAAAATCAAAGAAAAAGAAGTTGCCCACCGCCTGGAAATTGATGATCTGAAAAGCAGCCATGTTCGTGATATTGGTCGT<br>ATTGTTGAAGCACATGATCTGGCACTGCAGAAACTGATTAATAGCCAGCAGCTGGTTGATGCAGCACGTGTTTATGAAAAACTGACCAGCGAA<br>GGTGTTGAAGGTCGTCTGAGCAATCTGGATGATCCGCTGAAAACCAGCAGCCGTTATAGCCTGAGCCATGCAAGCACACCGGCACGTCGTACC<br>CTGCCTCCGACCACCGGTAGCGCAGTTAGCCTGTTTAGCGCAACCCCGAAAGGTAGCGATATTAAACTGGAAGGTGATGGTCTGCAGAGCGTT<br>TATCGTGCAAAATTTGCAGAAGGCACCAGCGTTAGCGTTAAACGTGAACGTGATGATGACCTGGTTACACCGGCAGCAAAAATTGTTAAAAAA<br>CGTACACCGCGTACGCCGAGCTATACCGCAGCAGAACGTGTTGCAGCAGCAAGC
	BA2246: TACTTCCAATCCATGCCGCAGGGTACAGTTAGCCG
	BA2249: TATCCACCTTTACTGTCAGCTTGCTGCTGCAACACGTT
pBA472	KKT4 coding sequence (4–1,383 bp: $KKT4^{2–461}$) with PacI and AscI, cloned into pBA310
	BA853: GATCTTAATTAAGGGAGACAATCCACTGTCTGT
	BA854: GATCGGCGCGCCCTATGGGCGAAACGGTCGGCGGA

Table S4. **Primers and synthetic DNA sequences used in this study *(Continued)***

To make	Primer or synthetic DNA sequences
pBA838	KKT4 coding sequence (4–1,935 bp: KKT4$^{2-645}$) with PacI and AscI, cloned into pBA310
	BA1311: GATCTTAATTAAGGGAGACAATCCACTGTCTGT
	BA1312: GATCGGCGCGCCTTAATCACGACTTATAGCGA
pBA1418	Following two PCR fragments were cloned into pEnT5-Y using XbaI and BamHI
	KKT4 3′ UTR targeting sequence with HindIII and NotI
	BA1964: GATCGATCAAGCTTCGGTTTGTGTGCGTATGGTACG
	BA1965: GATCGATCGCGGCCGCGGCCTTATCCACGCCTAAACCGTGC
	KKT4 coding sequence (201–1,935 bp) with R123E, K132E, R154E mutations with NotI and SpeI, amplified from pBA1407
	BA1968: GATCGATCGCGGCCGCTATGGGAGCAGCATCTTCTC
	BA1967: GATCGATACTAGTACCTGCTCCATCACGACTTATAGCGAAACG
pBA1518	Following two PCR fragments were cloned into pEnT5-Y using XbaI and BamHI
	KKT4 3′ UTR targeting sequence with HindIII and NotI
	BA1964: GATCGATCAAGCTTCGGTTTGTGTGCGTATGGTACG
	BA1965: GATCGATCGCGGCCGCGGCCTTATCCACGCCTAAACCGTGC
	Wild-type KKT4 coding sequence (201–1,935 bp) with NotI and SpeI
	BA1968: GATCGATCGCGGCCGCTATGGGAGCAGCATCTTCTC
	BA1967: GATCGATACTAGTACCTGCTCCATCACGACTTATAGCGAAACG
pBA1606	Following two PCR fragments were cloned into pEnT5-Y using XbaI and BamHI
	KKT4 3′ UTR targeting sequence with HindIII and NotI
	BA1964: GATCGATCAAGCTTCGGTTTGTGTGCGTATGGTACG
	BA1965: GATCGATCGCGGCCGCGGCCTTATCCACGCCTAAACCGTGC
	Wild-type KKT4 coding sequence (1–1,935 bp) with NotI and SpeI
	BA2127: GATCGATCGCGGCCGCATGGGAGACAATCCACTGTCTG
	BA1967: GATCGATACTAGTACCTGCTCCATCACGACTTATAGCGAAACG
pBA1610	To delete the region corresponding to 115–174 amino acids in the KKT4 gene with PCR from pBA1606. The PCR product was self-ligated with T4 polynucleotide kinase and T4 DNA ligase
	BA1670: GTTGTTGAGGAGAAAGAGAGCGCATTAGAGAAACACG
	BA1669: CCCTACATCGCTTTTGCTGTCACCACGCGAC

Table S4. **Primers and synthetic DNA sequences used in this study *(Continued)***

To make	Primer or synthetic DNA sequences
pBA925	Synthetic DNA sequence with XmaI and NheI, cloned into pACEBac2 to express SNAP-6HIS-3FLAG-KKT4 (codon optimized for expression in insect cells)
	CCCGGGATGGACAAGGACTGCGAGATGAAGCGTACCAC CCTGGACTCCCCACTGGGCAAGCTGGAACTGTCCGGTTGCGAGCAGGGCC TGC<br>ACCGTATCATCTTCCTCGGCAAGGGCACCTCCGCTGCTGACGCTGTG GAAGTGCCTGCTCCTGCTGCTGTGCTGGGTGGTCCTGAGCCTCTG<br>ATGCA GGCTACCGCTTGGCTGAACGCTTACTTCCACCAGCCCGAGGCTATCGAGG AATTCCCCGTCCCTGCTCTGCACCACCCCGTGTTCC<br>AGCAAGAATCCTTC ACCAGGCAGGTCCTGTGGAAGCTGCTGAAGGTGGTCAAGTTCGGCGAAGT GATCTCCTACTCCCACCTGGCTGCTCT<br>GGCTGGCAACCCTGCTGCTACCG CTGCTGTCAAGACCGCTCTGTCCGGAAACCCCGTGCCCATCCTGATCCCT TGCCACCGTGTGGTGCAA<br>GGCGACCTGGACGTCGGTGGTTACGAGGGTGG ACTGGCTGTGAAGGAATGGCTGCTGGCTCACGAGGGTCACCGTCTGGGAA AGCCTGGAC<br>TGGGT GGTAGCGGCGAGTTCAAGCTGGGCCTGATCAACCGT GGTTCTCATCACCATCATCACCACGCTGGAAAGGCTGGCGAATTCATGGA<br>CTACAAGGACGACGACGACAAGGATTACAAGGATGATGATGATAAGGACT ACAAGGACGACGATGATAAGGGTTCCGCTGGT GGCGACAAC<br>CCTCTGTCCGTCCTGCAGCAGCTGGCTTCCAACCCCCAGCTGATCACCCAGATGCAGGG TATCCTGGCTCTGCTGTCCCCTGGTCACGGCGA<br>CGGTGCTACTGGTGCTA GACCTGGTGGTGTCGGCCTCGAGCAGATCGCTGCTCTGTGCCCTCAGCCT CGTACCCTGCAGAACACCGCTACT<br>ACCGATATGGGTGCTGCTTCCTCCCT GATGGCTGGTGGCCTGAACGTCGAGCCTCCTCGTCAGCAGACCCGTTGGC CTTCTCCTGTGGCTA<br>CCGCTTCCGAGTCCTCCAACCTGCAGTCCCGTGGC GACTCCAAGTCCGACGTGGGAAAATACGGTGTCGTGTCCGTCGAGCGTTA CGAGCG<br>TCTGATGGCTCGTTACAAGGAACTCGAGAAGCAGTCCCACCGTC GTCAGGGCAAGCGTTCCGAACCTGTGGTGGACACCCAGCGTGTGCTGG<br>AC CTGGAAGAGGAAGTGGCTCGTCTGAAGCGTACCATCGGCCACTTGCAGGG TGTCGTGGAAGAGAAGGAATCCGCTCTGGAAAAGCACGC<br>TACCCAGCACA ACCTGGAAGTGCACGAGATGAAGAAGAACTACGAGCTGAAGATCAAGTCC CTGACCCAGACCCACGAGGCTGCTGTGCGC<br>AAGCTGGTGTCCGCTCAAGA GCTGGTCACCGCTGCTCGTAACTACCAGACCGCTGTGTGCGCTAACAACG TGGGCGGTGGCAACTCCGTGT<br>CTACCACCTCCGGTCAACCCCTGTCCAAC ACCGTGAACCACACCCGCGGACTGACCACCACCTCCTCCGGTTCCGGTCC CAACCAGCCTTA<br>CACCCTGCCTCACCCTGACGGCAACGCTTGGATGTCCG CTACTACCTCCGACGACCGTTCCGCTCCCGTGACCACCAAGAACTCCCAC TCC<br>GTGAAGCGCGAGCGCGAGGGTACTGTGTCTACTACCCCTACCCGTCC CCTGAAGAAGCGTAACCCCCGTACCCCCTCCTACACCGTGGCTGA<br>CCGTA TCTCCGAGACTGACGAGTACGTGAAGAAGGGTTCCCCTGTCGTCAAGAAG GAACTGAACTCCCCCACCGCTGAGGCCCAGCAGACT<br>TGCTTGGACGGCAT GGGTTACCAGCCTGCTGTGGGTTCCAACATCCTGCGTGCTCAGACCTCTT ACACCTCCGCTGCTTGCACCCTGGTCG<br>AGGCTGTGTCCAACTCCCGCCAG CAGTTCCAGCAGCAGCGTTCTGAGTCCGTGCAGATGCAGCCCCGTTCCGG TGCTGTGGCTCTGCGTCA<br>ACGTACCGGTGGACTGTCCCCCGTGGCTTCCT CCTCTGGTTCTGCTCTGATGGCCACCCCTTCCTCCCGTGGATTGCGTCGT CCTTTCCGT<br>CCTGTGTCCGGCGCTTCTTCCGCTGTCGGTGGTTCAACCCG TTCCCCATCCCCTGTGGACCCTAAGCGTGGTGCTGTGCAGCCTCGCTACT<br>TCATCACCACTTCCCTGACCGAGAAGGAACGCAACTCTGTGATGGAAGCC ATCCAAAAGCTGGGCCAGCGTGCTGTGCTGGTGGACAACAAG<br>GTGGACGA GATCCTGCCCCTGAACACCACCCACATCGTGCTGCGTGGTCCCCCTCGTT CCGTGAAGGCTCTGTGCGGTGTTGTGTCCTCAA<br>AGTGGCTGGTGCAGCCC TCCTACGTGTTCGACTCCCTGGGTGCTGGTTTCTGGCTGGACGAAGAGGT CGAGGGTGGACTGCGCTACTTCCC<br>CCCACCTCTGCGTTGCCAGCGTTTCC TGCTGACCATGCCTGAGGGCGTGGTCAAGACCATGCTGCAGCGTGTGGTC GAGTTCGGTGGTGGC<br>GAGGTCGTGGGCACCAAGCGTAACGGTTCCTCCAA CGACCAGGACGTGGTCGTGGTGTCCTCTGGCGACGAGCTGCTGCGTTTCG CTATCT<br>CCCGCGACTAAGCTAGC
pBA1351	Site-directed mutagenesis primers to make SNAP-6HIS-3FLAG-KKT4$^{R123E,\ K132E,\ R154E}$ from pBA925 (codon optimized for expression in insect cells)
	R123E
	BA1736: CGGTGTCGTGTCCGTCGAGGAGTACGAGCGTCTGATGGCTCGTTAC
	BA1737: CGAGCCATCAGACGCTCGTACTCCTCGACGGACACGACACCGTATTTTCCC
	K132E
	BA1738: GAGCGTCTGATGGCTCGTTACGAGGAACTCGAGAAGCAGTCCCACCGTCG
	BA1739: GTGGGACTGCTTCTCGAGTTCCTCGTAACGAGCCATCAGACGCTCGTA
	R154E
	BA1740: CGAACCTGTGGTGGACACCCAGGAGGTGCTGGACCTGGAAGAGGAAGTGG
	BA1741: CTTCCTCTTCCAGGTCCAGCACCTCCTGGGTGTCCACCACAGGTTCGGAACG

All primers are listed in the 5′ to 3′ direction.

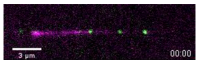

Video 1. **Wild-type KKT4 tracks with a disassembling microtubule tip.** TIRF movie of wild-type, ^{549}SNAP-tagged KKT4 (green) tracking with a depolymerizing, Alexa Fluor 488–labeled microtubule (magenta). KKT4 concentration was 3.5 nM. To induce microtubule depolymerization, free tubulin (10 µM) was washed out from the chamber. The video corresponds to the event in Fig. 5 A. It was recorded at 5 frames per second.

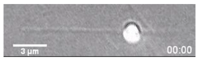

Video 2. **A bead decorated with wild-type KKT4$^{115–343}$ diffuses on the microtubule lattice and then undergoes disassembly-driven motion.** Microtubule depolymerization drives the movement of the bead toward the microtubule minus end. The contrast-enhanced video corresponds to the event in Fig. 5 B, which was recorded at 30 frames per second. The bead was decorated at 60 nM of KKT4.

Provided online is one table in Excel. Table S1 is a summary of optical trap–based bead motility assays and is related to Figs. 4 (F and G), 5 C, and S2.

References

Akiyoshi, B., and K. Gull. 2014. Discovery of unconventional kinetochores in kinetoplastids. *Cell.* 156:1247–1258. https://doi.org/10.1016/j.cell.2014.01.049

Buchan, D.W.A., F. Minneci, T.C.O. Nugent, K. Bryson, and D.T. Jones. 2013. Scalable web services for the PSIPRED Protein Analysis Workbench. *Nucleic Acids Res.* 41:W349-57. https://doi.org/10.1093/nar/gkt381

Gileadi, O., N.A. Burgess-Brown, S.M. Colebrook, G. Berridge, P. Savitsky, C.E.A. Smee, P. Loppnau, C. Johansson, E. Salah, and N.H. Pantic. 2008. High throughput production of recombinant human proteins for crystallography. *Methods Mol. Biol.* 426:221–246. https://doi.org/10.1007/978-1-60327-058-8_14

Hayashi, H., and B. Akiyoshi. 2018. Degradation of cyclin B is critical for nuclear division in *Trypanosoma brucei*. *Biol. Open.* 7:bio031609. https://doi.org/10.1242/bio.031609

Katoh, K., and D.M. Standley. 2013. MAFFT multiple sequence alignment software version 7: improvements in performance and usability. *Mol. Biol. Evol.* 30:772–780. https://doi.org/10.1093/molbev/mst010

Kelly, S., J. Reed, S. Kramer, L. Ellis, H. Webb, J. Sunter, J. Salje, N. Marinsek, K. Gull, B. Wickstead, and M. Carrington. 2007. Functional genomics in *Trypanosoma brucei*: a collection of vectors for the expression of tagged proteins from endogenous and ectopic gene loci. *Mol. Biochem. Parasitol.* 154:103–109. https://doi.org/10.1016/j.molbiopara.2007.03.012

Lupas, A., M. Van Dyke, and J. Stock. 1991. Predicting coiled coils from protein sequences. *Science.* 252:1162–1164. https://doi.org/10.1126/science.252.5009.1162

Nerusheva, O.O., and B. Akiyoshi. 2016. Divergent polo box domains underpin the unique kinetoplastid kinetochore. *Open Biol.* 6:150206. https://doi.org/10.1098/rsob.150206

Poon, S.K., L. Peacock, W. Gibson, K. Gull, and S. Kelly. 2012. A modular and optimized single marker system for generating *Trypanosoma brucei* cell lines expressing T7 RNA polymerase and the tetracycline repressor. *Open Biol.* 2:110037. https://doi.org/10.1098/rsob.110037

Waterhouse, A.M., J.B. Procter, D.M.A. Martin, M. Clamp, and G.J. Barton. 2009. Jalview Version 2--a multiple sequence alignment editor and analysis workbench. *Bioinformatics.* 25:1189–1191. https://doi.org/10.1093/bioinformatics/btp033
